# Chikungunya virus dissemination from the midgut of *Aedes aegypti* is associated with temporal basal lamina degradation during bloodmeal digestion

**DOI:** 10.1371/journal.pntd.0005976

**Published:** 2017-09-29

**Authors:** Shengzhang Dong, Velmurugan Balaraman, Asher M. Kantor, Jingyi Lin, DeAna G. Grant, Nicole L. Held, Alexander W. E. Franz

**Affiliations:** 1 Department of Veterinary Pathobiology, University of Missouri, Columbia, Missouri, United States of America; 2 Electron Microscopy Core Facility, University of Missouri, Columbia, Missouri, United States of America; Centers for Disease Control and Prevention, UNITED STATES

## Abstract

In the mosquito, the midgut epithelium is the initial tissue to become infected with an arthropod-borne virus (arbovirus) that has been acquired from a vertebrate host along with a viremic bloodmeal. Following its replication in midgut epithelial cells, the virus needs to exit the midgut and infect secondary tissues including the salivary glands before it can be transmitted to another vertebrate host. The viral exit mechanism from the midgut, the midgut escape barrier (MEB), is poorly understood although it is an important determinant of mosquito vector competence for arboviruses. Using chikungunya virus (CHIKV) as a model in *Aedes aegypti*, we demonstrate that the basal lamina (BL) of the extracellular matrix (ECM) surrounding the midgut constitutes a potential barrier for the virus. The BL, predominantly consisting of collagen IV and laminin, becomes permissive during bloodmeal digestion in the midgut lumen. Bloodmeal digestion, BL permissiveness, and CHIKV dissemination are coincident with increased collagenase activity, diminished collagen IV abundance, and BL shredding in the midgut between 24–32 h post-bloodmeal. This indicates that there may be a window-of-opportunity during which the MEB in *Ae*. *aegypti* becomes permissive for CHIKV. Matrix metalloproteinases (MMPs) are the principal extracellular endopeptidases responsible for the degradation/remodeling of the ECM including the BL. We focused on *Ae*. *aegypti* (Ae)MMP1, which is expressed in midgut epithelial cells, is inducible upon bloodfeeding, and shows collagenase (gelatinase) activity. However, attempts to inhibit AeMMP activity in general or specifically that of AeMMP1 did not seem to affect its function nor produce an altered midgut escape phenotype. As an alternative, we silenced and overexpressed the *Ae*. *aegypti* tissue inhibitor of metalloproteinases (AeTIMP) in the mosquito midgut. AeTIMP was highly upregulated in the midgut during bloodmeal digestion and was able to inhibit MMP activity *in vitro*. Bloodmeal-inducible, midgut-specific overexpression of AeTIMP or its expression via a recombinant CHIKV significantly increased midgut dissemination rates of the virus. Possibly, AeTIMP overexpression affected BL degradation and/or restoration thereby increasing the midgut dissemination efficiency of the virus.

## Author summary

The biological nature of the midgut escape barrier in insects for arthropod-borne viruses has been a mystery for decades. Here we show that the basal lamina (BL) surrounding the mosquito midgut acts as a barrier for chikungunya virus, an alphavirus, which has emerged in the New World hemisphere around three years ago. The barrier became permissive for the virus during digestion of a viremic bloodmeal inside the midgut lumen. Concurrent with BL permissiveness, we observed that collagen IV, a major component of the BL became temporally degraded while the BL was visibly damaged. Based on previous findings, we hypothesized that matrix metalloproteinases such as *Ae*. *aegypti* (Ae)MMP1 may be involved in BL degradation. We confirmed that recombinant AeMMP1 exhibited strong gelatinase activity, which was profoundly reduced when recombinant AeMMP1 interacted *in vitro* with the recombinant *Ae*. *aegypti* tissue inhibitor of metalloproteinases (AeTIMP). When transgenically overexpressing AeTIMP in an attempt to temporally inhibit general MMP activity in the mosquito midgut, we observed that the dissemination efficiency of chikungunya virus became significantly increased, while its midgut infection was not affected. It is possible that AeTIMP overexpression affected BL degradation/restoration permitting increased quantities of virus to escape from the midgut.

## Introduction

*Aedes aegypti* is the primary vector for important human pathogenic arboviruses such as dengue virus (*Flaviviridae*; *Flavivirus*; DENV1-4), chikungunya virus (*Togaviridae*; *Alphavirus*; CHIKV), and Zika virus (*Flaviviridae*; *Flavivirus*; ZIKV) [1,2]. Following acquisition of a viremic bloodmeal from a human, viruses such as CHIKV enter the midgut lumen of the female mosquito along with the bloodmeal (reviewed in: [3]). In contrast, a sugarmeal is deposited into the crop and therefore does not enter the midgut. Within a few hours, before formation of the peritrophic matrix and at the onset of bloodmeal digestion, virus needs to enter and infect the midgut epithelial cells where it starts replicating. In the epithelium, *de novo* synthesized virions accumulate at the basal lamina (BL) surrounding the midgut to disseminate to secondary tissues such as hemocytes, fat body, nerve tissue, and eventually the salivary glands. The latter need to be infected before virus can be released along with saliva during probing to infect another host. The pore size exclusion limit of the BL (9–12 nm) is too small for arbovirus virions (50–80 nm in diameter) to pass through [4]. Thus, it has been postulated that the midgut BL structure needs to be temporally modified to enable virions to disseminate from the midgut. Based on current knowledge, viruses cannot actively penetrate the midgut BL in order to disseminate, which suggests that traversing the BL by a virus requires the activation of an endogenous mechanism of the host/vector that causes BL modification [5,6]. Acquisition of a bloodmeal is a natural process in mosquitoes causing the midgut tissue along with its BL to expand multi-fold of its typical size in a sugarfed mosquito. We observed that in *Ae*. *aegypti*, viruses such as CHIKV disseminate from the midgut during the time window during which bloodmeal digestion takes place [7]. In insects such as *Drosophila*, the BL is a sheet-like network of extracellular matrix (ECM) components predominantly composed of (non-fibril) collagen IV and laminin and synthesized during early development by hemocytes and the fat body [8]. In mammals, the formation of the BL is initiated by a self-assembling laminin network [9–11]. A second network is then formed by collagen IV to provide mechanical strength. Finally, the laminin and collagen IV networks are linked together by nidogen and perlecan [12,13]. Experimental evidence suggests that collagen IV is fundamental for the maintenance of integrity and function of basement membranes under conditions of increasing mechanical demands, but dispensable for deposition and initial assembly of components for which laminin seems to be sufficient [14]. In *Drosophila*, BL associated collagen IV is a heterotrimeric protein consisting of two conserved subunits, α1-like and α2-like encoded by the genes *Cg25C* and *Vkg*, respectively [15]. Human collagen IV is composed of three (heterotrimeric) α1-like and α2-like subunits, which are encoded by six different genes, *alpha 1*-*alpha 6* [16–17].

Matrix metalloproteinases (MMPs) are known as the principal metalloproteases that cleave components of the ECM during tissue remodeling/wound healing [18–20]. In humans, the BL components collagen IV and laminin are degraded by gelatinases such as HuMMP2 and HuMMP9, and by stromelysins such as HuMMP3 and HuMMP10 [20]. Mammalian MMPs can also produce specific substrate-cleavage fragments with independent biological activities, for example the release of ECM-bound growth factors (insulin growth factor, fibroblast growth factor). Further, MMPs can act as important regulators of extracellular tissue signaling networks [21,22]. *D*. *melanogaster* possesses two MMPs, Dm1-MMP and Dm2-MMP, which are both involved in tissue remodeling [23]. Typically, Dm1-MMP mediates BL degradation; however, when under the control of the JNK signaling pathway during wound healing, Dm1-MMP is required for assembly and repair of the BL, which likely is achieved by cleaving existing BL fragments in order to insert new molecules [24]. MMPs are typically present in the extracellular matrix as zymogens with a typical domain structure consisting of N-terminal propeptide, (central) catalytic, and C-terminal hemopexin domains. MMP activity is tightly regulated at several levels: gene expression, protein activation via cleavage of the ~10–13 kDa N-terminal propeptide domain, inhibition via binding to a tissue inhibitor of metalloproteinases (TIMP), and finally protein degradation [25,26]. Recently, we described the MMPs in *Ae*. *aegypti* as a family of proteases that could be potentially involved in BL modification/remodeling during virus dissemination from the midgut [27]. In particular, two of the nine AeMMPs, AeMMP1 and AeMMP2, were found to be associated with midgut tissue and they also responded to the presence of a bloodmeal containing or not containing CHIKV. AeMMP1 contains the propeptide, catalytic, and hemopexin domains and appears to be membrane-bound. AeMMP2 lacks a canonical propeptide domain but contains catalytic and hemopexin domain structures.

Originally, the four mammalian TIMPs (TIMP1-4) have been described as specific inhibitors of MMPs, but subsequent studies showed that human TIMP3 (HuTIMP3) can also bind to the disintegrin-metalloproteinases (ADAM) and ADAMTS (ADAM with thrombospondin motifs) to block their activities [28]. TIMP-mediated inhibition of an MMP occurs via binding of the N-terminal structure of TIMP to the catalytic cofactor of the target MMP [29,30]. In mammals, TIMPs also have biological activities that are independent of metalloproteinase interactions [31]. These include effects on cell growth and differentiation, cell migration, antiangiogenesis, anti- and pro-apoptosis, and synaptic plasticity. HuTIMPs can interact directly with specific cell surface receptors to induce cellular responses. HuTIMP1, for example, can activate the MAPK pathway via receptor binding [32]. Based on comparative genomic analyses, it has been assumed that insect genomes generally encode only a single TIMP, which is most similar to HuTIMP3 [33–35]. Studies with *D*. *melanogaster* (Dm)TIMP showed that it can inhibit the two endogenous DmMMPs, particularly Dm1-MMP [35]. *In vitro*, DmTIMP was also able to inhibit the activities of HuMMP1-3, human ADAM17, and to a lesser extent human ADAM10 [36].

In this study, we show that in sugarfed mosquitoes, the non-expanded midgut BL constitutes a barrier for CHIKV. The BL became permissive for the virus during bloodmeal ingestion causing expansion of the midgut tissue. Tissue expansion in bloodmeal-containing midguts coincided with temporal collagen IV degradation/dimishment and significantly increased collagenase activities. Transgene or virus-mediated overexpression of AeTIMP, which had the capacity to inhibit AeMMP activity *in vitro*, resulted in an increased viral midgut dissemination phenotype.

## Results

### In sugarfed mosquitoes, the midgut BL is not permissive for CHIKV although in bloodfed mosquitoes, the BL becomes permissive for the virus

**Fig 1:**
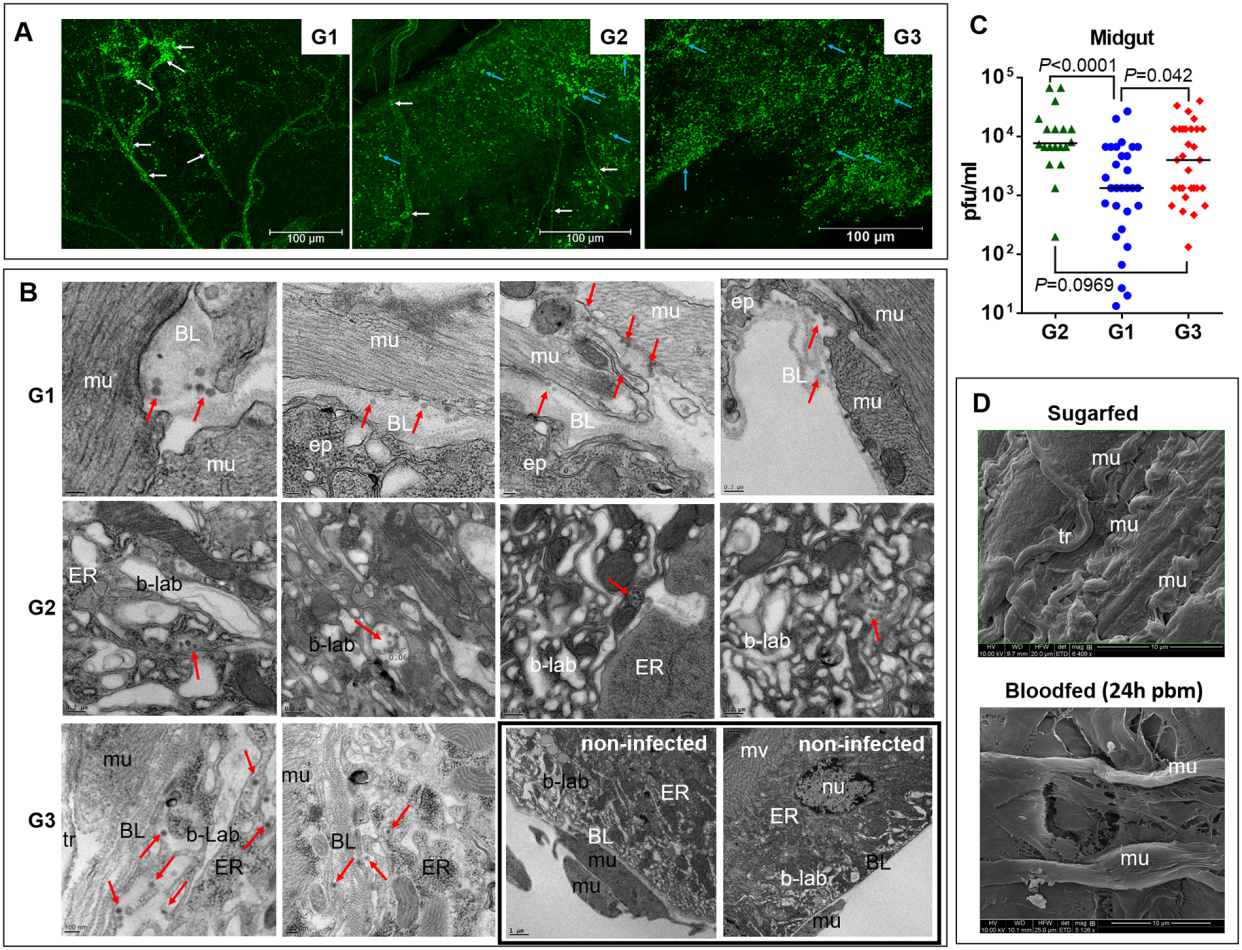
The midgut basal lamina (BL) is a barrier for CHIKV dissemination, which becomes permissible when a bloodmeal is present in the midgut. Group 1 mosquitoes (**G1**): CHIKV (10^5^ pfu/ml) was intrathoracically injected into females, which were then maintained on sugar diet. Midguts were analyzed at 4 days post-injection. Group 2 mosquitoes (**G2**): CHIKV was intrathoracically injected into females, which received a bloodmeal the following day. Midguts were analyzed at 3 days post- bloodfeeding. Group 3 mosquitoes (**G3**): CHIKV was orally acquired along with a bloodmeal and midguts were analyzed at 4 days post-infection (dpi). **(A)** Immunofluorescence assay based detection of CHIKV antigen (green) in midgut tissues of G1, G2, and G3 mosquitoes at 4 dpi using a CHIKV-specific monoclonal antibody. White and blue arrows indicate examples of viral antigen associated with tracheal cells and midgut epithelial cells, respectively. Bars = 100 μm. **(B)** Ultrastructural TEM images showing cross sections of midgut tissue of mosquitoes at 4 days post-injection with CHIKV (G1: BL surrounding the epithelium in proximity to midgut-associated muscles; G2: epithelium with basal labyrinth and ER) and 4 days post-bloodfeeding (G3: BL surrounding the epithelium in proximity to midgut-associated muscles). Red arrows indicate virions; scale bars are indicated on each image. Each image represents a different midgut sample. Note: in G1, there were no virions visible within the epithelium. The two images within the black frame (not belonging to G3) were taken from non-infected samples and provide an overview of the subcellular organization of a midgut. Abbreviations: BL, basal lamina; ep, epithelium; mu, muscle; b-lab, basal labyrinth; ER, endoplasmatic reticulum; mv, microvilli; nu, nucleus; tr, tracheal cell. **(C)** CHIKV titers (pfu)/ml) in midgut tissue of G1, G2, and G3 mosquitoes at 4 dpi as determined by plaque assays in Vero cells. *P*-values were determined by the Mann-Whitney U-test. **(D)** Ultrastructural SEM images of the surface structure of midguts dissected from sugarfed and bloodfed HWE females. Abbreviations: mu, muscle; tr, tracheal cell.

CHIKV (strain: 37997; 10^5^ plaque-forming units (pfu)/ml) was intrathoracically injected into bloodfed and sugarfed HWE females. Immunofluorescence assays using a CHIKV-specific monoclonal antibody showed that following intrathoracic injection, the virus did not enter and infect the midgut epithelium of those females that had received a sugarmeal instead of a bloodmeal the following day (Fig 1A, G1). Instead, the virus predominantly infected the tracheal cells surrounding the midgut tissue. Transmission electron microscopy (TEM) images demonstrated that in sugarfed mosquitoes, intrathoracically injected CHIKV was tethered to the BL at 4 days post-injection; however, virions were not observed within the epithelial cells (Fig 1B, G1). In contrast, intrathoracically injected CHIKV infected midgut epithelial cells and replicated within the cells in those females that had received a virus-free bloodmeal the following day (Fig 1A and 1B, G2). Ultrastructural analysis of several midgut samples showed virus in the basal labyrinth of the midgut epithelium in close proximity to the ER at 1 day post-(virus-free) bloodmeal (pbm) (see red arrows), indicative of *de novo* synthesized virions (Fig 1B, G2). Acting as a positive control, orally acquired CHIKV (via artificial bloodmeal) readily infected midgut epithelial cells and was detectable in those at 4 days post-infection (dpi) (Fig 1A and 1B, G3). Accordingly, median CHIKV titers were significantly higher in midguts of those mosquitoes that had acquired the virus orally (G3) or via injection followed by acquisition of a bloodmeal (G2) than in midguts of sugarfed mosquitoes, which had received the virus via injection (G1) (Fig 1C). Scanning electron microscopy (SEM) images of the midgut surface structure revealed that in midguts of bloodfed females at 24 h pbm, the BL appeared to be thinly stretched with outer BL layer(s) in close proximity to the latitudinal and longitudinal muscles being shredded and substantially degraded (Fig 1D). In contrast, the midgut surface of sugarfed mosquitoes looked relaxed and 'wrinkled', with no BL damage being visible.

All these observations allow the conclusion that the BL is a barrier for CHIKV infection and that the presence of a bloodmeal in the midgut causes the BL to become permissive for virus to infect secondary tissues. As injected virus could not efficiently enter the midgut epithelium of sugarfed mosquitoes, virus infection became restricted to the tracheal cells surrounding the midgut.

### BL-associated collagen IV is diminished during bloodmeal digestion

**Fig 2:**
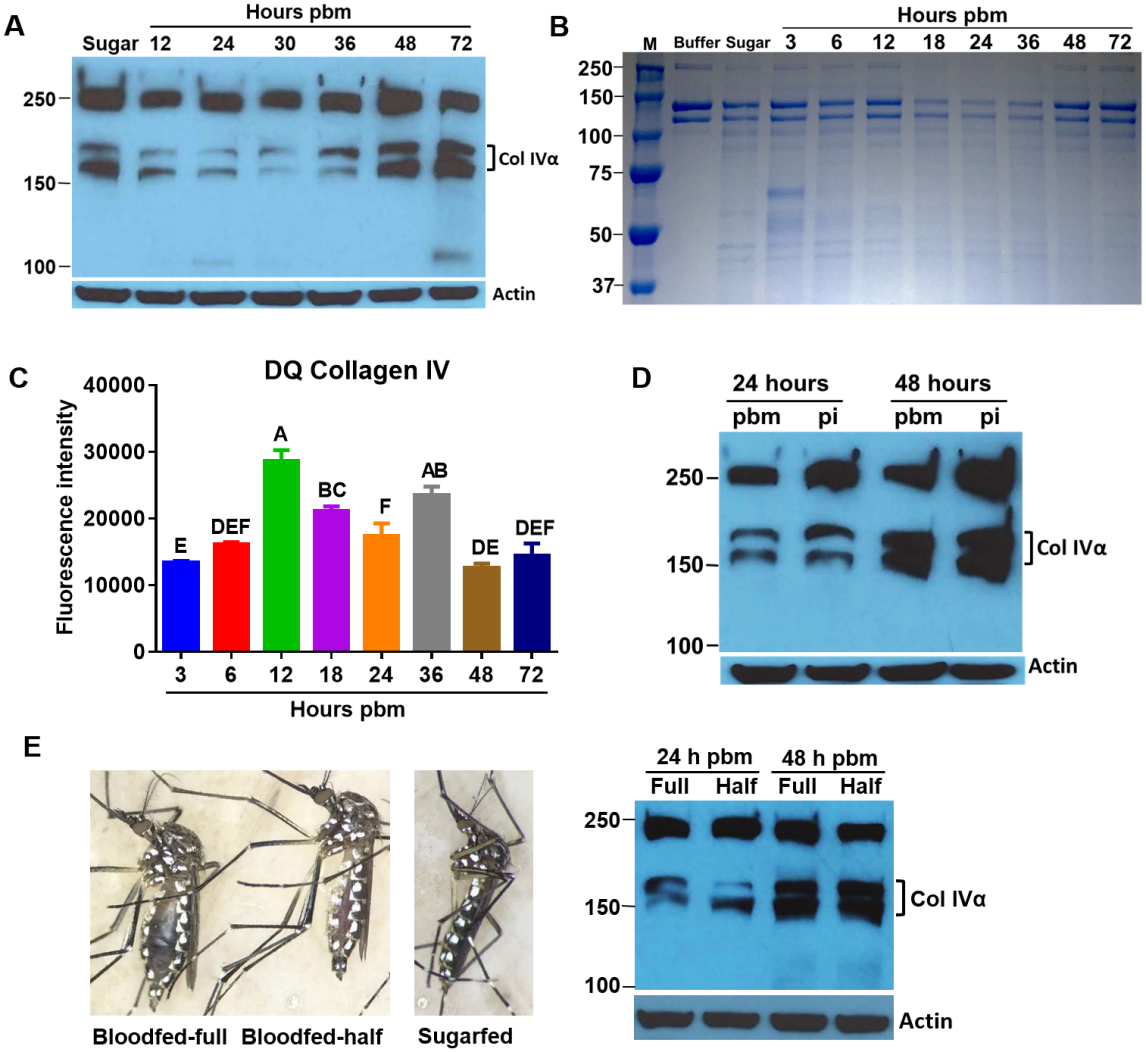
Midgut BL associated collagen IV is diminished during bloodmeal digestion, which coincides with increased collagenase activity in the midgut between 12–36 h pbm. **(A)** Detection of collagen IV in midguts of mosquitoes at different time points post-bloodmeal (pbm) and in sugarfed mosquitoes (Sugar) by Western blot using polyclonal antibodies generated against human collagen IV; two α chains of collagen IV (Col IVα) are detected. β-actin was detected as a loading control. Standard molecular sizes are indicated. **(B)** SDS-PAGE for the detection of human placenta collagen I, which was incubated with lysates prepared from mosquito midguts at different time points pbm. **(C)** *In vitro* activity assay to detect collagenase IV activity among lysates prepared from mosquito midguts at different time points pbm. Mean values with standard deviations from three independent experiments are shown. Different letters indicate significant differences based on one-way analysis of variance (ANOVA) followed by Tukey's multiple comparisons test (* at *p* ≤ 0.05). **(D)** Detection of collagen IV by Western blot in midguts of mosquitoes that had received a non-infectious or a CHIKV-containing bloodmeal at 1 and 2 days pbm/pi using polyclonal antibodies generated against human collagen IV. Images shown in panels A, B, D, are representative examples of repeated experiments. **(E)** Images of *Ae*. *aegypti* HWE females at 1 day pbm. The female on the left is fed to repletion ("full"), the one in the center is partially-fed ("half"), and the one on the right is sugarfed (no meal has entered the midgut). Detection of collagen IV in midguts of females, which were bloodfed to repletion ("full") or partially-fed ("half") at 1 and 2 days pbm by Western blot using polyclonal antibodies against human collagen IV; two α chains of collagen IV (Col IVα) were detected. β-actin was detected as a loading control.

We investigated whether the change in midgut BL permissiveness during bloodmeal acquisition/digestion was reflected by a change in the abundance of collagen IV, which is a core com*p*onent of the BL. Midgut collagen IVα (~160–180 kDa) was clearly less abundant in midguts of females at 12–36 h pbm as shown by Western blots using polyclonal antibodies generated against human collagen IV (Fig 2A). At 48 h pbm and later time points, collagen IV abundance recovered to levels observed in midguts of sugarfed mosquitoes. Incubation of human placenta collagen I with lysates prepared from midguts at different time points post-bloodmeal showed a similar time window during which collagen I was diminished, indicating that the midgut lysates possessed collagenase activity between 12 and 36 h pbm (Fig 2B). Accordingly, similarly prepared midgut lysates showed significantly increased collagenase IV activity *in vitro* between 12 and 36 h pbm (Fig 2C). The observed period of collagenase activity in the mosquito midgut concurred with the period of bloodmeal digestion during which CHIKV dissemination took place (see also: [7]). However, the presence of CHIKV had no obvious effect on collagen IV degradation at 24 or 48 h post-infection via oral acquisition (pi) (Fig 2D). Interestingly, diminished detection of midgut collagen IV was also observed at 24 h pbm in those mosquitoes which were not fed to repletion but had ingested only a partial bloodmeal instead (Fig 2E). This indicates that variable meal volumes were sufficient to trigger the process.

Despite the ability to detect collagenase activity in the midgut of bloodfed females using different approaches, it was not possible in several attempts to manipulate midgut collagen IV degradation/abundance *in vivo* via supplementation of bloodmeals with collagenase or MMP inhibitors (such as GM6001) or intrathoracic injection of these compounds (S1A, S1B, S1C and S1D Fig). This prevented us from demonstrating whether CHIKV would be able to disseminate when collagen IV is not diminished/degraded. Importantly, however, CHIKV was not able to infect midgut epithelium cells when it was intrathoracically injected into sugarfed mosquitoes in which collagen IVα was not visibly degraded (Fig 1A and 1B; Fig 2A).

### AeMMP1, possessing collagenase (gelatinase) activity, is highly expressed during bloodmeal digestion and can be inhibited by AeTIMP

**Fig 3:**
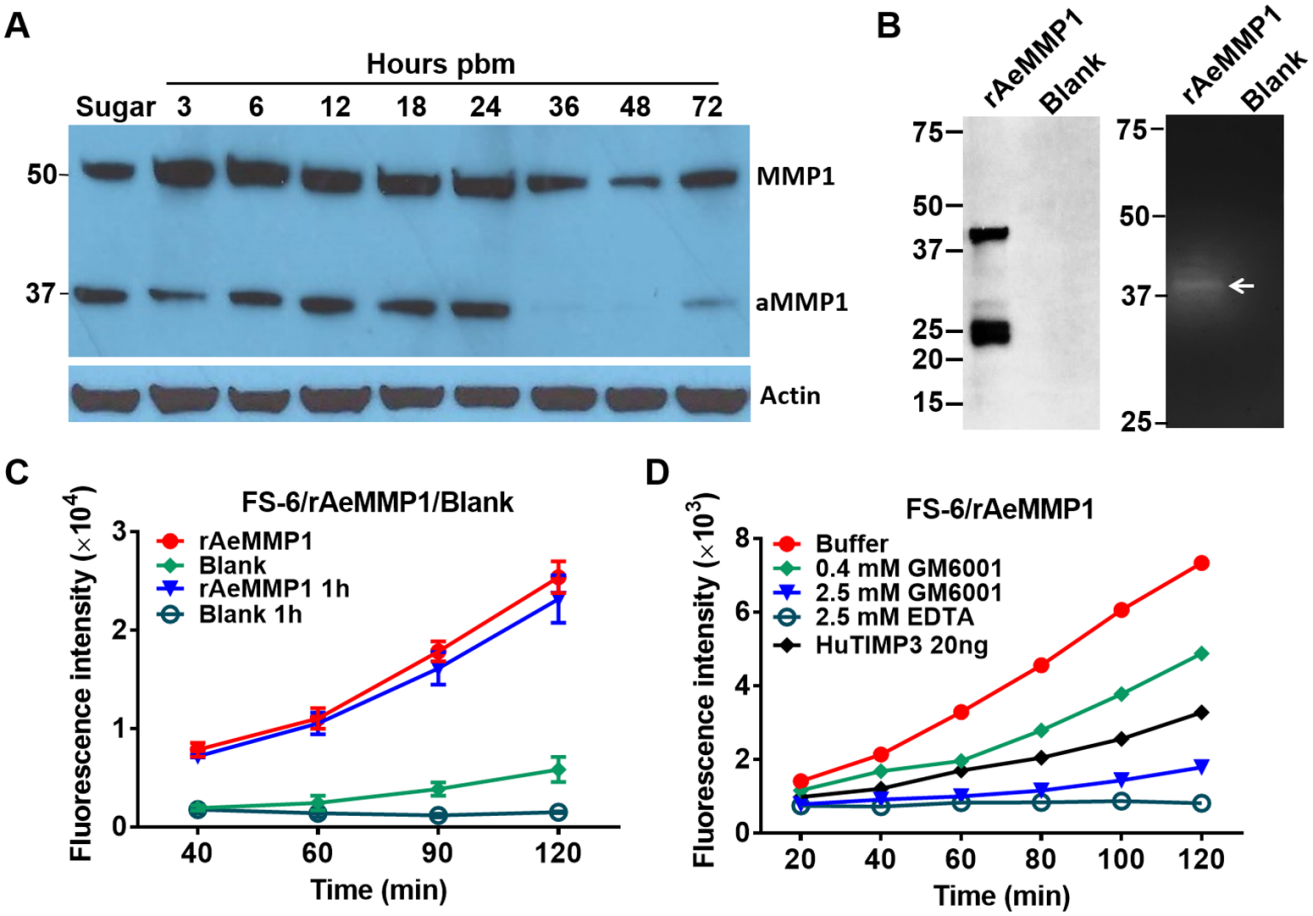
The activated form of AeMMP1 is detectable in midguts during the early phase of bloodmeal digestion and activated, recombinant AeMMP1 exhibits enzymatic activity *in vitro*. **(A)** Detection of AeMMP1 in mosquito midguts at different time points pbm by Western blot using polyclonal antibodies pAb-mmp1-2. (aMMP1 = active form of AeMMP1). β-actin was detected as a loading control. (**B)** Western blot (left) and gelatin zymography (right) to detect the catalytically active form of recombinant, *Drosophila* S2 cell expressed (r)AeMMP1. White arrow indicates catalytically active form of rAeMMP1. (**C)** Kinetics of rAeMMP1 activity in an *in vitro* MMP activity assay using FS-6 as substrate. Twenty ng of rAeMMP1 were preincubated with 1 mM proMMP activator 4-aminophenylmercuric acetate (AMPA) or reaction buffer for 1 h before start of the activity assay. Blank, rAeMMP1: cell culture medium transfected with "empty" plasmid vector and rAeMMP1 expression plasmid, respectively; blank 1 h, rAeMMP1 1 h: medium was preincubated for 1 h with 1 mM AMPA before FS-6 substrate incubation. (**D**) Kinetics of GM6001, EDTA or HuTIMP3-mediated inhibition of rAeMMP1. Twenty ng of HuTIMP3, 0.4 mM GM6001, 2.5 mM GM6001, or 2.5 mM EDTA were preincubated with 10 ng of rAeMMP1 at RT for 2 h followed by addition of FS-6 substrate. The fluorescence intensity was measured every 20 min.

Recently, we showed that two of the nine MMPs of *Ae*. *aegypti*, AeMMP1 and AeMMP2, were active in midgut tissue and responded to the presence of a bloodmeal [27]. AeMMP2 was associated with tracheal cells surrounding the midgut, whereas AeMMP1 was highly expressed in epithelial cells, the site at which CHIKV infection occurs. This prompted us to focus our functional studies on AeMMP1. The zymogen (50 kDa) and the active form of AeMMP1 (37 kDa) lacking its propeptide domain were highly expressed between 3 and 24 h pbm (Fig 3A) concurring with the time period during which collagen IV was less detectable (Fig 2A). Similar to what we had recently observed [27], the catalytically active form of AeMMP1 was barely detectable in midguts between 36 and 72 h pbm (Fig 3A). Typically, most of the bloodmeal has been digested by 48 h post-acquisition allowing the midgut tissue to contract and the BL to rebuild. It is possible that diminished AeMMP1 activity or presence during this period benefits subsequent BL rebuilding.

*In vitro*, we confirmed the catalytic activity of recombinant (r)AeMMP1 produced in *Drosophila* S2 cells. Following expression in S2 cells, rAeMMP1 (lacking the signal peptide) generated two major band signals with molecular masses of ~40 and 25 kDa, which were recognized by pAb (polyclonal antibodies)- Aemmp1-2 (antigenic region in the hemopexin domain; Fig 3B and S2 Fig) but not by pAbAemmp1-1 (antigenic region near C-terminus outside the hemopexin domain; S2 Fig), or by an antibody specific to the recombinant, C-terminal V5 tag. The ~40 kDa band signal likely represented the active form of rAeMMP1 lacking the propeptide domain as treatment with proMMP activator 4-aminophenylmercuric acetate (AMPA) had no effect on rAeMMP1 activity (Fig 3C). This was further confirmed by gelatin zymography in which the 40 kDa band signal represented the rAeMMP1 protein with catalytic activity, whereas the band signal corresponding to a mass of 25 kDa, possibly representing the cleaved hemopexin domain (minus its C-terminus), showed no catalytic activity (Fig 3B). In generic MMP activity assays using the FS-6 substrate, the S2 cell generated rAeMMP1 showed strong MMP activity (Fig 3C and 3D). Furthermore, treatment with the broad spectrum MMP inhibitor GM6001, metal chelator EDTA, or recombinant human TIMP3 (HuTIMP3) strongly reduced recombinant rAeMMP1 activity *in vitro* (Fig 3D).

Our experiments indicate that AeMMP1 has collagenase (gelatinase) activity and therefore could be involved in the midgut BL remodeling process. To support this hypothesis, we attempted to modulate the expression of AeMMP1 in the mosquito. Transient silencing of *Aemmp1* reduced its transcript abundance in whole-body mosquitoes by ~70% at 48 h post-injection (S3A Fig) but unexpectedly, AeMMP1 protein levels in midguts and CHIKV infections of carcasses (whole mosquito bodies from which the midguts had been removed) were not affected at 1 day pbm (= 3 days post-injection) (S3B Fig). Consequently, collagen IV degradation levels at 1 day pbm in midguts and titers of orally acquired CHIKV at 2 dpi in carcasses of *Aemmp1* dsRNA injected females were not different from those of the *Luc* dsRNA injected control (S3C and S3D Fig). We then decided to inhibit MMP activity in the mosquito via overexpression of the MMP antagonist TIMP as an alternative approach to reveal any effects of MMP impairment on CHIKV dissemination.

### Recombinant AeTIMP_V5_ can inhibit MMP activity

Initially, we compared the amino acid sequence of AeTIMP with that of *Ae*. *albopictus* (Aa)TIMP and human (Hu)TIMP3 to reveal their similarities. The 213 aa AeTIMP shares >91% aa identity with AaTIMP but only 29% aa identity with HuTIMP3, which is most similar to arthropod TIMPs [36,37] (S4A Fig). AeTIMP and AaTIMP have six conserved cysteine residues in their N-terminal and eight cysteine residues in their C-terminal subdomains in common, forming three and four disulfide bonds, respectively, which are involved in protein structure formation [37]. HuTIMP3 and AeTIMP contain their six cysteine residues of the N-terminal domain, which is involved in MMP interaction, at similar positions [36]. However, in the C-terminal domain, HuTIMP3 only has six cysteine residues instead of eight.

**Fig 4:**
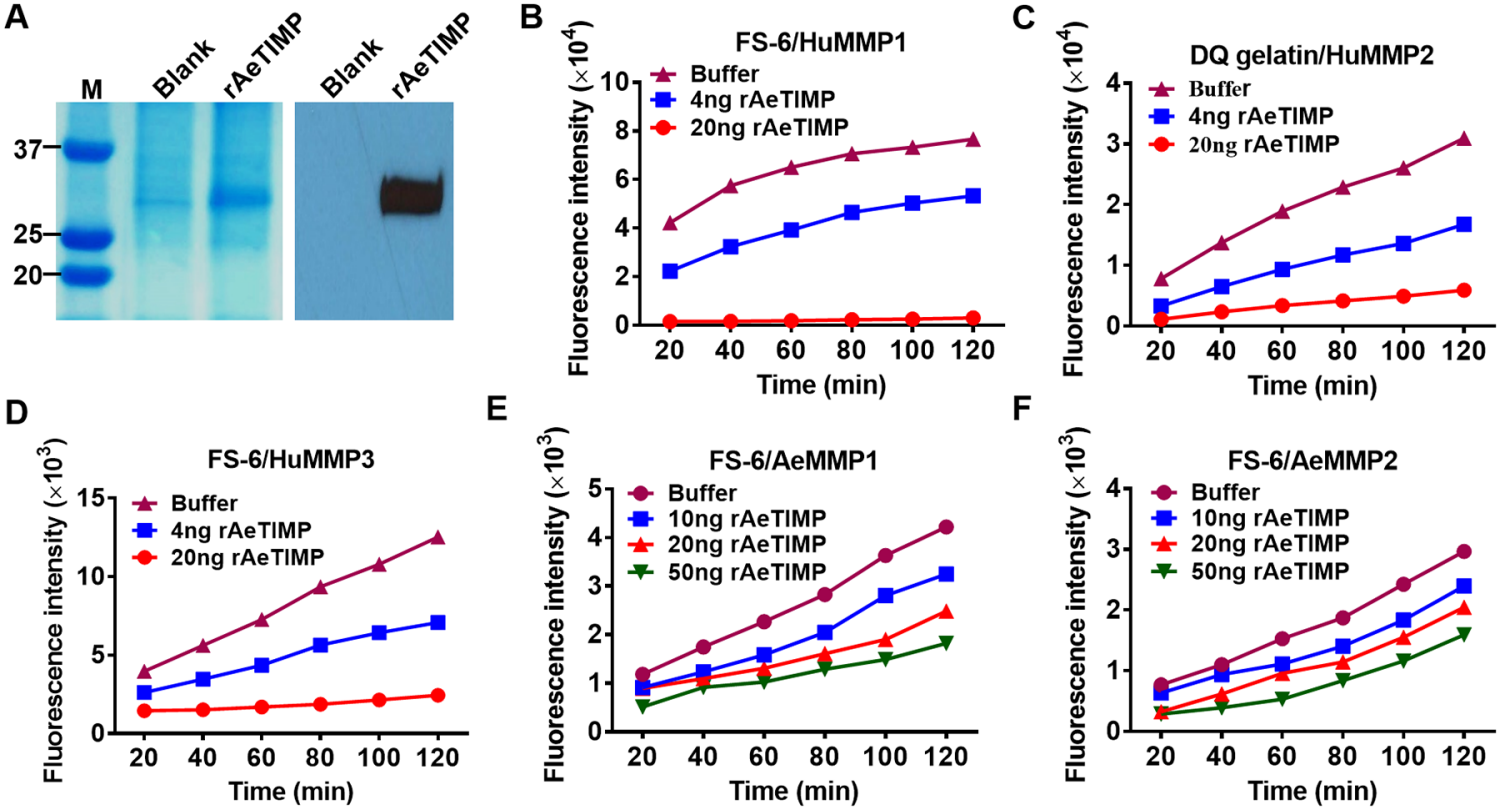
Recombinant AeTIMP inhibits MMP activity *in vitro*. (**A**) SDS-PAGE (left) showing a ~30 kDa band signal of purified rAeTIMP following its expression in HEK293T cells; Western blot (right) of purified rAeTIMP detected by a monoclonal anti-His-tag antibody. Blank, rAeTIMP: HEK293T cells were transfected with "empty" plasmid or AeTIMP expression plasmid, respectively. (**B**) Kinetics of rAeTIMP-mediated inhibition of human MMP1, (**C**) human MMP2, (**D**) human MMP3. Four ng or 20 ng of rAeTIMP, or buffer were incubated with 20 ng of each MMP at RT for 2 h, followed by addition of FS-6 or DQ gelatin (human MMP2 only) substrate and incubation for an additional 2 h. Fluorescence intensity was measured every 20 min. (**E**) Kinetics of rAeTIMP-mediated inhibition of rAeMMP1 and (**F**) rAeMMP2. Ten ng of rAeMMP1 or rAeMMP2 were incubated with 10 ng, 20 ng or 50 ng rAeTIMP at RT for 2 h, followed by addition of FS-6 substrate. Graphs in panels B-F are representative examples of multiple experiments.

We expressed AeTIMP in human embryonic kidney (HEK)293T cells, which produced a specific band signal with a molecular mass of around 30 kDa as confirmed by Western blot analysis using a His-tag specific monoclonal antibody (Fig 4A). Recombinant (r)AeTIMP proved to be functional as it was able to strongly inhibit the activity of three human MMPs, HuMMP1, HuMMP2, and HuMMP3 in activity assays *in vitro* using FS-6 or DQ gelatin (for HuMMP2) as substrates (Fig 4B, 4C and 4D). rAeTIMP exhibited a weaker inhibitory effect on rAeMMP1 and rAeMMP2 (the latter of which was included in the experiment for comparison) (Fig 4E and 4F). In similar assays, HuTIMP3 showed robust inhibition of HuMMP2 and HuMMP3 (S4B and S4C Fig) and of rAeMMP1 (Fig 3D). Importantly, rAeTIMP *per se* did not show any MMP-like catalytic activity *in vitro*, confirming that the observed reduced MMP activities were caused by interactions between rAeTIMP and MMPs leading to the inhibition of the latter (S4D Fig).

### Overexpression of AeTIMP in midguts of mosquitoes from a recombinant CHIKV increases the midgut dissemination efficiency of the virus

**Fig 5:**
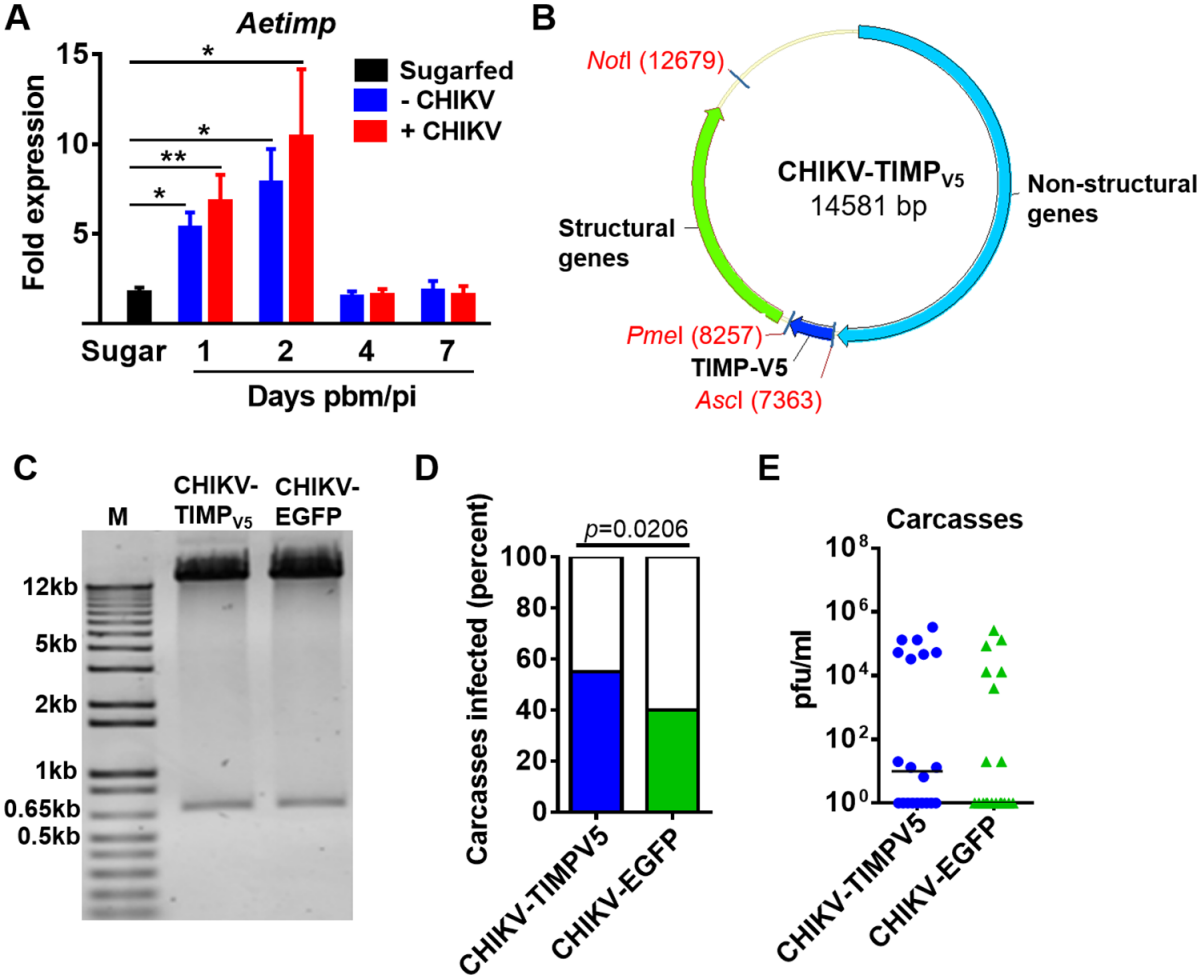
Expression profile of AeTIMP in mosquito midguts and its overexpression via a recombinant CHIKV leading to increased carcass infection rates. **(A)** *Aetimp* expression profile in midguts of HWE mosquitoes, which had received a non-infectious or a CHIKV-containing bloodmeal at 1, 2, 4, and 7 days pbm/pi as detected by qRT-PCR. Mean values with standard deviation (SD) from three independent experiments are shown. Significance between the sugarfed control and bloodfed/CHIKV-infected samples was determined by Student's *t*-test (* at *p* ≤ 0.05, ** at *p* ≤ 0.01). **(B)** Schematic map of the recombinant CHIKV-TIMP_V5_ construct based on CHIKV strain 2006 LR-OPY1, which harbors the A226V amino acid change in E1. The viral cDNA clone was engineered to have its duplicated subgenomic promoter for gene-of-interest expression positioned upstream of the structural genes. **(C)** Restriction enzyme digest of CHIKV-TIMP_V5_ and CHIKV-EGFP plasmids with *Asc*I and *Pme*I showing gene-of-interest inserts and remaining full-length cDNAs of the viral clones. **(D)** Carcass infection rates and **(E)** virus titers in carcasses of mosquitoes at 4 dpi, following acquisition of bloodmeals containing 10^7^pfu/ml CHIKV-TIMP_V5_ or CHIKV-EGFP. Fisher’s exact test was used to determine *p*-values in **(D)**. In **(E)**, each data point represents the CHIKV titer of a single carcass. *P*-values were determined by the Mann-Whitney U-test. Black bars indicate medians.

Since rAeTIMP inhibited MMP activity *in vitro*, we assessed whether manipulating AeTIMP expression levels in the mosquito midgut would affect CHIKV dissemination from this organ. Endogenous *Aetimp* expression was significantly upregulated (up to 8-fold at 2 day dpi; *p* ≤ 0.01 or ≤ 0.05) in midguts at 1 and 2 days pbm in comparison to its expression in midguts of sugarfed females (Fig 5A). Transient silencing of *Aetimp* significantly reduced transcript abundance (by ~3-fold) in those mosquitoes which had received a non-infectious bloodmeal at 2 days post-dsRNA injection in comparison to the negative controls, mosquitoes injected with *egfp* dsRNA or PBS (S5A Fig). However, transient *Aetimp* silencing had no significant effects on CHIKV dissemination rates and virus titers in carcasses at 2 dpi (S5B Fig). Overexpression of AeTIMP_V5_ using a recombinant CHIKV cDNA clone (strain: LR2006 OPY1, "CHIKV- AeTIMP_V5_"; [38]; Fig 5B, 5C, 5D and 5E) resulted in significantly increased carcass infection rates in orally infected HWE females at 4 dpi compared to the carcass infection rates observed in HWE females orally infected with the same CHIKV strain expressing EGFP ("CHIKV-EGFP") instead of AeTIMP_V5_ (Fig 5D). Virus titers of carcasses were similar at 4 dpi for both CHIKV-TIMP_V5_ and CHIKV-EGFP infected mosquitoes (Fig 5E). The results indicate that CHIKV-mediated expression of AeTIMP_V5_ enabled the virus to disseminate from the midgut of *Ae*. *aegypti* with a higher efficiency than CHIKV-mediated expression of a reporter gene.

### Generation of transgenic mosquitoes overexpressing AeTIMP_V5_ in the midgut of bloodfed females

**Fig 6:**
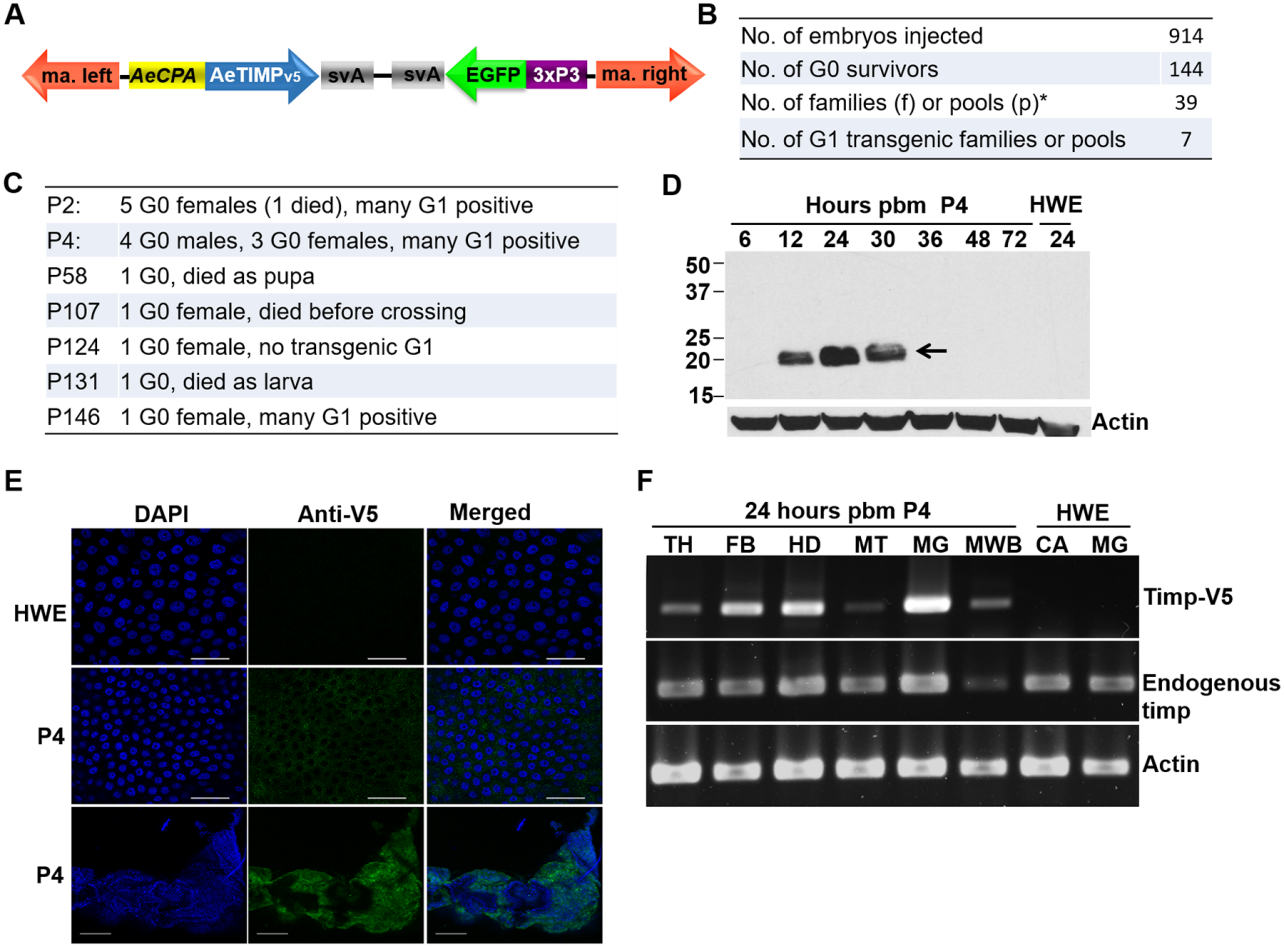
Transgene-mediated overexpression of AeTIMP_V5_ in midguts of bloodfed mosquitoes of line P4. **(A)** Diagram of the *mariner Mos1* TE construct to overexpress AeTIMP_V5_ under control of the bloodmeal-inducible, midgut-specific *AeCPA* promoter. Abbreviations: ma. left, ma. right = left, right arm of *mariner Mos1*; *AeCPA* = *Ae*. *aegypti carboxypeptidase A* promoter; svA, polyadenylation signal of simian virus 40 VP1 gene; 3xP3 = eye tissue-specific promoter. (**B, C**) Data of germline transformation experiment and resulting transgenic G_0_ founders. **(D)** Detection of AeTIMP_V5_ in midguts of transgenic line P4 at different time points post-bloodmeal by Western blot using a V5 tag specific monoclonal antibody. β-actin was detected as a loading control. Arrow indicates AeTIMP_V5_ protein. **(E)** Immunofluorescence assay for the detection of transgenic AeTIMP_V5_ antigen (green) in midgut tissue of P4 mosquitoes at 24 h pbm. V5 tag antigen was detected using a specific monoclonal antibody. Nuclei were stained with DAPI (blue). Bars = 40 μm (first two rows) and 200 μm (third row). **(F)** RT-PCR detection of transgene-expressed AeTIMP_V5_ and endogenous AeTIMP in various tissues of P4 mosquitoes at 24 h pbm. Abbreviations: TH = thorax; FB = fat body; HD = head tissue; MT = malpighian tubules; MG = midgut; MWB = whole-body males; CA = carcass.

To confirm that overexpression of AeTIMP_V5_ increased CHIKV dissemination efficiency from the mosquito midgut, we generated transgenic *Ae*. *aegypti* expressing AeTIMP_V5_ from the *AeCPA* promoter (Fig 6A). The AeTIMP_V5_ expression cassette was inserted into the *mariner Mos1* transposable element (TE) [39,40]. Nine-hundred-and-fourteen preblastoderm HWE embryos were co-injected with the *mariner Mos1* TE encoding plasmid and the transposase encoding helper plasmid (Fig 6B). The resulting 144 G_0_ survivors were outcrossed to HWE mosquitoes in 39 pools, seven of which produced transgenic G_1_ offspring based on EGFP eye marker expression originating from the TE. Curiously, four of the seven transgenic lines (P58, P107, P124, P131) were founded by single individuals, which died or were unable to produce any transgenic offspring (Fig 6C). G_0_ founders of P2 and P146 were also females, whereas P4 G_0_ founders consisted of males and females. AeTIMP_V5_ expression was strongest in line P4 in which the recombinant protein was clearly detected in midguts of females at 12–30 h pbm (Fig 6D). This line was selected for further studies and immunofluorescence assays confirmed AeTIMP_V5_ expression in the entire midgut epithelium at 24 h pbm (Fig 6E). Further, in line P4, AeTIMP_V5_ was not restricted to the midgut but was also moderately detectable in fatbody (FB), head tissue (HD) and weakly detectable in the thorax (TH), the malphigian tubules (MT) and in males (MWB) (Fig 6F). Genotypic characterization of line P4 revealed a single TE integration at nt position 1,211,152 of supercontig 1.342 (chromosome 2q) (S6A Fig).

### Transgenic overexpression of AeTIMP in midguts of mosquitoes increases the midgut dissemination efficiency of CHIKV and enhances infection with DENV4

**Fig 7:**
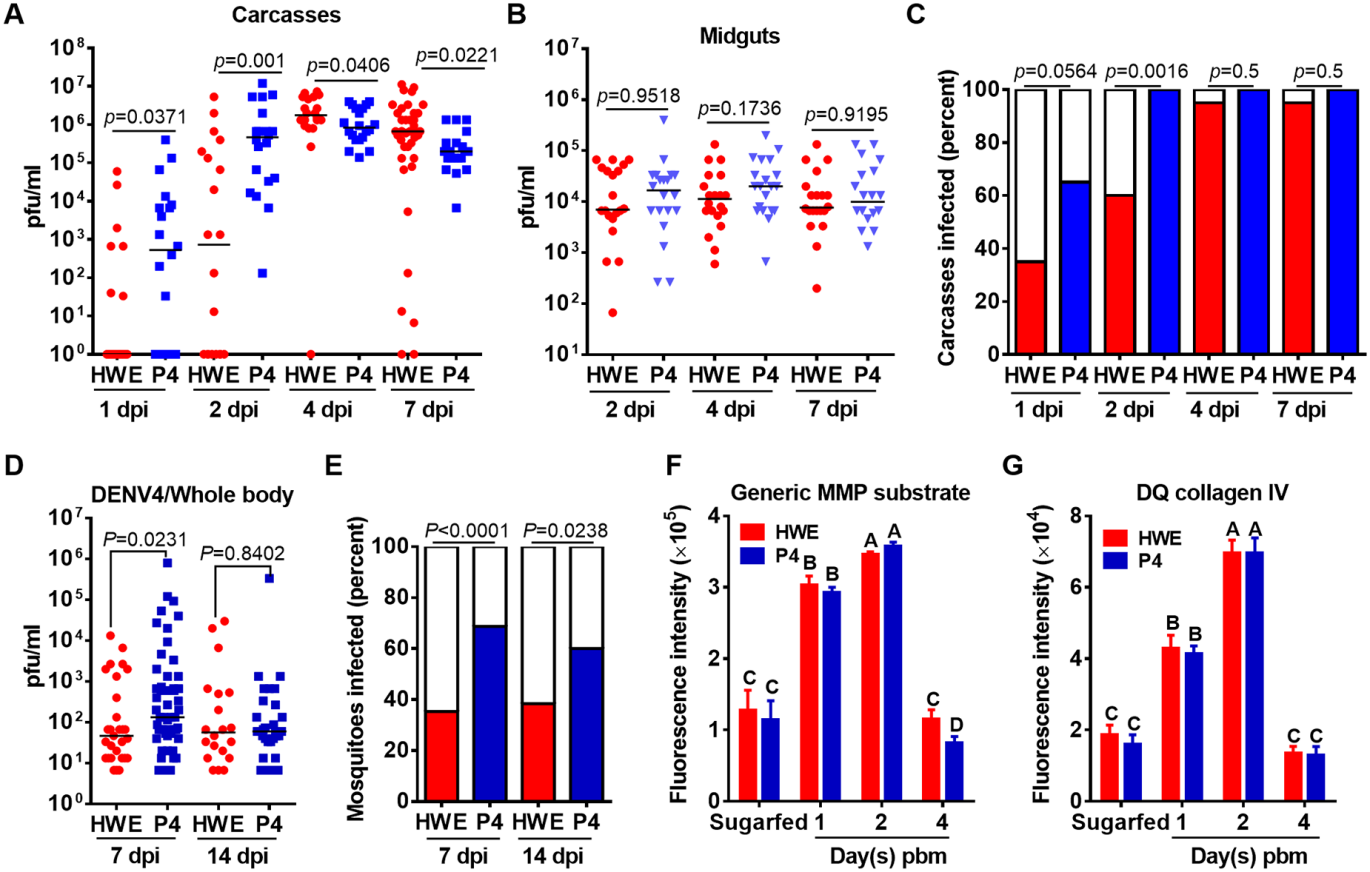
Transgene-mediated overexpression of AeTIMP_V5_ in midguts of bloodfed P4 mosquitoes increases CHIKV dissemination efficiency and DENV4 whole-body titers, infection rates. **(A)** CHIKV titers of individual HWE and (G_4_) P4 carcasses and **(B)** midguts at 1, 2, 4, 7 dpi as analyzed by plaque assays in Vero cells. Each data point represents the CHIKV titer of a single midgut or carcass. Black bars indicate medians. Only infected mosquitoes were included in the statistical analysis using the Mann-Whitney U-test to determine *p*-values. **(C)** CHIKV infection rates among carcasses of HWE and P4 mosquitoes at 1, 2, 4, 7 dpi as analyzed by plaque assays in Vero cells. Fisher’s exact test was used to determine *p*-values. **(D)** DENV4 (strain: H-241) titers of individual HWE and P4 mosquitoes and **(E)** DENV4 infection rates of HWE and P4 mosquitoes at 7 and 14 dpi as analyzed by plaque assays in BHK21 cells. Statistical analyses were performed as in A-C. Graphs in panels A-E are representative examples of repeated experiments. Comparison of **(F)** generic MMP and **(G)** collagenase IV activities between midguts of HWE and P4 mosquitoes at 1, 2, 4 days pbm using generic MMP and DQ collagen IV substrates. Different letters indicate significant differences based on one-way analysis of variance (ANOVA) followed by Tukey's multiple comparisons test (* at *p* ≤ 0.05).

Following oral challenge of (G_4_) P4 mosquitoes with CHIKV (titer in the bloodmeal: 10^7^ pfu/ml), virus titers at 1 and 2 dpi (Fig 7A) and virus infection rates at 2 dpi (Fig 7C) were significantly increased in the carcasses of P4 females compared to carcasses of the similarly challenged HWE control. At later time points (4 and 7 dpi) when AeTIMP_V5_ was no longer overexpressed in the mosquito midgut, CHIKV infection rates in P4 carcasses were similar to those of HWE. Importantly, midgut infection rates and titers for CHIKV were similar in HWE and P4 mosquitoes over a seven-day time course (Fig 7B, S6B and S6C Fig) suggesting that AeTIMP_V5_ overexpression did increase CHIKV dissemination from the midgut but not the viral infection/replication efficiency in the midgut. Oral challenge with DENV4 (strain: H-241; titer in the bloodmeal: 5x10^6^ pfu/ml) resulted in significantly increased virus titers in P4 mosquitoes at 7 dpi and in significantly increased infection rates in P4 mosquitoes at 7 and 14 dpi as compared to the HWE control (Fig 7D and 7E). Both, P4 and HWE mosquitoes exhibited similar patterns of MMP and collagenase IV activities over a four-day time course, with the exception of day 4 (pbm), when generic MMP activity was significantly lower in P4 females than in HWE females (Fig 7F and 7G). In both lines, MMP and collagenase IV activities were significantly increased at 1 and 2 days pbm when compared to 4 day pbm or the sugarfed control.

Our data from repeated experiments show that overexpression of AeTIMP in midgut tissue of *Ae*. *aegypti* led to significantly increased CHIKV dissemination rates and also to significantly increased whole-body mosquito infection rates with DENV4. Thus, AeTIMP overexpression in the mosquito midgut promoted the infection of two unrelated arboviruses even though collagenase IV activity in midguts of line P4 was similar to that of the non-transgenic HWE strain during the four-day observation period. Furthermore, we did not observe any difference between HWE and line P4 regarding their level of BL degradation as shown by SEM imaging (S7 Fig). BL degradation in close proximity to muscle tissue was clearly visible in midguts of bloodfed HWE or P4 mosquitoes at 24 h pbm but not in the sugarfed control (see also: Fig 1D). Although virus dissemination was significantly enhanced in P4 mosquitoes, the overall tissue modifications in the transgenic line leading to the increased virus dissemination phenotype may have been rather subtle and not easily distinguishable from those of the HWE control among the limited number of EM specimens that were examined.

## Discussion

In this study, we describe novel processes involved in CHIKV dissemination from the midgut of *Ae*. *aegypti* in an attempt to shed light on the molecular principles that define the mosquito MEB for arboviruses. We demonstrated that in sugarfed females, the midgut BL was not permissible for CHIK virions which associated with the BL but did not cross it during four days post-intrathoracic injection of the virus. This changed when there was a bloodmeal present in the midgut at 24 h post-intrathoracic CHIKV injection as under this condition, *de novo* synthesis of virus was observed 24 h later within the epithelial cells indicating that injected virions had crossed the BL. In parallel, midgut-associated tissue damage was clearly visible in midguts of bloodfed mosquitoes in which the outer layer(s) of the BL in close proximity to the muscles were shredded. This was never observed in the relaxed midgut tissue of sugarfed mosquitoes. It may well be possible that CHIK virions require binding proteins or structures such as cyto-skeleton associated actin to move virions towards the BL within the infected cells [41,42]. However, even when assuming that those required proteins were absent in our injected virus preparations, it would still not explain why the virus was impeded at the BL and could not cross it, unless the BL in sugarfed mosquitoes does constitute a barrier for the virus. Similarly, Smith and colleagues [43] reported that in sugarfed *Ae*. *taeniorhynchus*, intrathoracically injected VEEV infected numerous secondary tissues such as gut-associated muscles, nerve tissue, hemocytes, fat body, salivary glands, and the intussuscepted foregut but not the midgut epithelium. This suggests that our observation might not be restricted to *Ae*. *aegypti* and CHIKV but may apply to other mosquito-arbovirus interactions as well.

This study together with our recent work indicates that CHIKV disseminates from the midgut through the BL during bloodmeal digestion but not via tracheal cells [7]. This contrasts with earlier investigations suggesting that arboviruses might use the tracheal cell/tracheole route to disseminate from the midgut [44–46]. In our previous experiments, orally acquired CHIKV infected midgut tracheal cells at a time point when the virus was already detectable in tissues outside the midgut [7]. In sugarfed mosquitoes, however, intrathoracically injected CHIKV became increasingly concentrated in midgut-associated tracheal cells over time as the virus seemed to be unable to enter the midgut epithelium through the BL.

One reason for the overall phenomenon could be that the BL surrounding the midgut epithelial tissue of mosquitoes acts as a molecular sieve to regulate the passage of molecules [47]. Due to its small pore size exclusion limit, the BL constitutes a principal barrier for virions [4]. For virions to pass through the BL, its pore size exclusion limit needs to be enlarged, at least temporarily. Increased BL permissiveness could be achieved via hydrolysis of BL collagen IV (and laminin) due to the activities of specific proteinases [11,12,48]. The proteinase activity affecting collagen IV structure and abundance could be triggered by midgut tissue overstretching as a result of meal ingestion, where stretch receptors may act as sensors [49]. In another study performed by our group, we showed that CHIKV orally acquired along with a saline meal (consisting of PBS) or a protein meal (consisting of BSA) instead of a bloodmeal productively infected the mosquito midgut and efficiently disseminated from the organ to secondary tissues [50]. Thus, bloodmeal-specific ingredients do not seem to be required for the virus to disseminate from the midgut and neither do mosquitoes need to be fed to repletion to trigger midgut BL collagen IV diminishment/degradation (as shown in this study). This supports the hypothesis that sensors associated with the midgut detect the presence of variable meal volumes in the midgut lumen thereby inducing enzymatic degradation rather than purely mechanical tearing of the collagen network. Previous observations (including a parallel study which is not part of this work) also suggest that CHIKV dissemination from the midgut occurred during a relatively narrow "window-of-opportunity", which lasted from ~24 h to 32 h post-oral acquisition of the infectious bloodmeal [7]. Thus, we did not extend the experiment to 7 days post-injection/post-bloodmeal, because we believe that by 7 days post-oral acquisition, the vast majority of CHIK virions has already disseminated from the midgut.

Our studies so far have not revealed whether or not the virus plays an active role in the dissemination process. Nevertheless, the window-of-opportunity for CHIKV dissemination was coincident with diminished BL collagen IV, which in turn correlated with significantly increased collagenase IV activity.

Although we have repeatedly made these concurrent observations an obvious limitation of our study is our inability to prove cause and effect by demonstrating that BL collagen IV degradation was a prerequisite for CHIKV dissemination. We tried to inhibit collagen IV degradation using different chemical inhibitors, but none of them showed any effect. As a consequence, these inhibitors did not dramatically affect CHIKV dissemination. To our knowledge, there are no reports showing successful inhibition of collagenase activity *in vivo* in any insect species. Thus, although we repeatedly observed virus dissemination coincident with BL degradation after bloodmeal ingestion, diminished collagen IV detection, and increased collagenase activities, it cannot be excluded that collagen modification may not be necessary for the viral dissemination from the midgut. Solving this problem will require additional, extensive studies.

In vertebrates, MMPs and ADAMTSs are the most important enzymes responsible for extracellular matrix (ECM) degradation and remodeling [51]. Similar to MMP1 of vertebrates, Dm1-MMP1 which is orthologous to AeMMP1 [27], can degrade mammalian fibronectin and type IV collagen and the enzyme's degrading activity can be completely blocked by generic MMP inhibitors such as EDTA and BB-94 [26,52]. Importantly, Stevens and Page-McCaw [24] discovered that after tissue injury, secreted Dm1-MMP facilitated restoration of continuous tissue by remodeling the BL, promoting cell elongation and actin cytoskeletal reorganization, and activating extracellular signal-regulated kinase signaling. The authors suggested that Dm1-MMP promoted assembly of collagen IV into the BL by cleaving the existing basement membrane in order to insert new molecules, a step that may be also required for temporal matrix expansion such as during bloodmeal ingestion by mosquitoes.

Our activity assays showed that rAeMMP1 was able to degrade gelatin and cleave an MMP substrate *in vitro*. This substrate cleavage activity could be inhibited by EDTA, the general MMP inhibitor GM6001, or rAeTIMP. Together, these observations are a strong indication that AeMMP1 possesses metalloproteinase activity. It was not possible to block AeMMP1 activity chemically or via gene silencing *in vivo* in order to reveal what effects diminished activity of the proteinase could have on BL degradation and/or CHIKV dissemination. In contrast, transient silencing of the MMP1 homologue in *Anopheles gambiae* caused a profound reduction in protein abundance resulting in an impaired phenotype [33]. Possibly, the putatively membrane-bound AeMMP1 [27] is a relatively stable protein that is maintained as a zymogen for extended periods of time despite protein production being temporarily cut off at the transcriptional and translational levels [53]. As a consequence, our observations so far do not allow us to conclude whether AeMMP1 is responsible for collagen IV degradation/diminishment during bloodmeal digestion or for collagen IV remodeling/restoration. Based on our data, both scenarios seem possible. Since AeMMP1 peak activity in midgut tissue concurred with collagen IV degradation/diminishment during bloodmeal digestion, it is possible that AeMMP1 could be involved in BL degradation during midgut tissue expansion as a consequence of bloodmeal ingestion. During the late phase of the bloodmeal digestion process (36–48 h pbm), active AeMMP1 is diminished (see also: [27]), which may stop any further collagen IV degradation and instead enable BL restoration. Assuming that AeMMP1 is responsible for BL restoration at the end of the bloodmeal digestion phase (rather than BL degradation at the beginning of bloodmeal ingestion/digestion), this could explain why AeTIMP overexpression leads to an increased dissemination efficiency of CHIKV. Enhanced AeTIMP expression would result in an increased inhibition of MMPs including AeMMP1 thereby preventing efficient BL restoration. Delayed or inefficient BL restoration would then leave the tissue in a 'leaky' state for the virus. However, we were unable to reveal any obvious differences in midgut collagen degradation levels between mosquitoes in which AeTIMP was overexpressed and control mosquitoes. Therefore, any effects on midgut collagen structure may have been rather subtle.

In mammals, TIMPs were reported to inhibit the activities of MMPs and ADAMs [28,29,31]. Recombinant DmTIMP showed inhibitory activities against MMP1 and MMP2 from *Drosophila* and MMP1-3, ADAM17, and ADAM10 from humans [25,36]. Although DmTIMP overexpression did not result in a visible phenotype, DmTIMP knockout resulted in a dysfunctional phenotype [54]. *In vitro*, rAeTIMP inhibited the activities of several HuMMPs and less efficiently that of AeMMP1 (or AeMMP2), whereas purified HuTIMP3 was able to inhibit the activity of rAeMMP1. Thus, rAeTIMP appeared to be functional but may exhibit a weaker affinity for rAeMMP1 than for the HuMMPs. As the number of MMPs can vary from two in *Drosophila* to nine in *Ae*. *aegypti* [27], the single AeTIMP may have a variable binding affinity for each MMP. However, when comparing these binding affinities, it needs to be kept in mind that these observations were primarily based on *in vitro* experiments to see whether there was any level of interaction detectable between the two protein types. Under *in vivo* conditions, actual affinity levels may look different than those observed *in vitro*. At any rate, similar to a previous study by Page-McCaw [23] we showed that rAeTIMP was able to cross-inhibit MMPs from different animal kingdoms, demonstrating that there is a structural conservation between HuTIMP3 and AeTIMP on the one hand and HuMMPs and AeMMP1 on the other. Thus, it seems likely that AeMMP1 is regulated, at least in part, by AeTIMP though the interaction may be relatively weak by comparison. Considering that vertebrate TIMPs are multifunctional proteins predominantly involved in ECM modification, it cannot be excluded that the effect of AeTIMP causing enhanced virus dissemination may be independent of its interaction with midgut associated AeMMPs. Under this scenario, the AeTIMP overexpression phenotype may have been caused by a direct interaction between AeTIMP and ECM surface molecules affecting permissiveness of the midgut BL. Previously, direct binding of HuTIMPs to ECM receptors has been described, which is independent of any interaction between the inhibitors and metalloproteinases [31,32,55]. However, in insects similar direct interactions between ECM and TIMP have not been reported to date.

In summary, our observations suggest that the MEB for arboviruses in mosquitoes is associated with structural changes of the BL. Bloodmeal ingestion associated with midgut tissue expansion leads to structural changes of the BL as exemplified by a temporary reduction in collagen IV abundance and visible shredding of the BL while arboviruses efficiently disseminate from the midgut. Assuming that collagen IV modification involves enzymatic processes, we show that AeMMP1, which is upregulated during bloodmeal digestion possesses collagenase (gelatinase) activity *in vitro* and can be inhibited by AeTIMP. Overexpression of AeTIMP in the mosquito midgut significantly enhances the midgut dissemination efficiency of CHIKV. Our study brought repeatedly and concurrently observed events into context. However, due to the complexity of the system, we have not yet been able to unambiguously establish a functional interrelationship. This work provides the foundation for further, detailed investigations to delineate the mechanism underlying MEB, which may involve numerous additional proteins with proteolytic and/or signaling functions.

## Material and methods

### Mosquitoes

*Ae*. *aegypti* Higgs White Eye (HWE), an eye-pigment deficient strain was reared and maintained at 28°C under 75–80% relative humidity and a 12h light/12h dark cycle in a BSL2 insectary. For colony maintenance, mosquitoes received artificial bloodmeals consisting of defibrinated sheep blood (Colorado Serum Company, Denver, CO).

### Transmission (TEM) and scanning (SEM) electron microscopy

Mosquito midguts were dissected at different time points and fixed in 100 mM sodium cacodylate buffer, pH 7.35 (Sigma Aldrich, St. Louis, MO) containing 2% paraformaldehyde and 2% glutaraldehyde. Each midgut was oriented and suspended in HistoGel (Thermo Scientific, Kalamazoo, MI). Samples were rinsed with 100 mM sodium cacodylate buffer, pH 7.35 containing 130 mM sucrose. Secondary fixation was performed using 1% osmium tetroxide (Ted Pella, Inc. Redding, CA) in 100 mM sodium cacodylate buffer followed by incubation in a Pelco Biowave Microwave Processing System (Ted Pella, Inc.) operated at 100 Watts for 1 min. Specimens were incubated at 4°C for 1 hour, then rinsed with cacodylate buffer and finally with distilled water. En bloc staining was performed using 1% aqueous uranyl acetate followed by incubation at 4°C overnight. Using the Pelco Biowave system, a graded dehydration series (per exchange, 100 Watts for 40 sec) was performed; first with ethanol then followed by acetone. Finally, dehydrated specimens were infiltrated with Epon resin (250 Watt for 3 min) and polymerized at 60°C overnight. Using an ultramicrotome (Ultracut UCT, Leica Microsystems, Wetzlar, Germany) equipped with a diamond knife (Diatome, Hatfield PA), 85 nm ultra-thin sections were prepared. Imaging was performed using a JEOL JEM 1400 transmission electron microscope (JEOL, Peabody, MA) at 80 kV equipped with a Gatan Ultrascan 1000 CCD acquisition camera (Gatan, Inc, Pleasanton, CA).

For SEM, midguts were dissected from HWE mosquitoes and fixed in 2% paraformaldehyde, 2% glutaraldehyde containing 100 mM sodium cacodylate buffer pH 7.35. Fixed tissues were rinsed with 100 mM sodium cacodylate buffer, pH 7.35 containing 130 mM sucrose. Secondary fixation was performed using 1% osmium tetroxide (Ted Pella, Inc.) in cacodylate buffer using a Pelco Biowave) operated at 100 Watts for 1 minute. Specimens were incubated at 4°C for 1 hour, then rinsed with cacodylate buffer and further with distilled water. Using the Pelco Biowave, a graded dehydration series (per exchange, 100 Watts for 40 sec) was performed using ethanol. Samples were dried using the Tousimis Autosamdri 815 Critical Point Dryer (Tousimis, Rockville, MD), and then sputter coated with 10 nm of platinum using an EMS 150T-ES Sputter Coater. Images were acquired with a FEI Quanta 600F scanning electron microscope (FEI, Hillsboro, OR).

### Transgene construction and generation of transgenic mosquitoes

Using a midgut cDNA library as template, the coding sequence of *Aetimp* was PCR amplified using forward and reverse primers containing *Not*I and *Sac*II restriction sites, respectively. The reverse primer also encoded the sequence for the V5 tag (S1 Table). Following double-digestion with *Not*I and *Sac*II, the *Aetimp*_*V5*_ cDNA was inserted into plasmid pSLfa1180fa-AeCPA/svA [39,40]. The resulting pSLfa1180fa-AeCPA/AeTIMP_V5_/svA expression cassette (Fig 6A) was digested with *Asc*I and inserted into the pMos-3xP3/EGFP/svA TE plasmid vector.

For germline transformation, the recombinant pMos1 TE plasmid was co-injected with the *mariner Mos1* transposase encoding helper plasmid into preblastoderm embryos of *Ae*. *aegypti* HWE as previously described [39,40]. Micro-injected mosquito eggs were maintained and hatched as described [39,40]. Each surviving G_0_ male was outcrossed to 10 virgin HWE females. Five G_0_ females were pooled and outcrossed to one HWE male. Progeny larvae of these crosses (G_1_) were screened for EGFP expression in their eyes using a Leica MZ 10F fluorescent stereo microscope (Wetzlar, Germany) equipped with an EGFP-specific filter set. Transgenic G_1_ mosquitoes were outcrossed to the HWE recipient strain and their progeny (G_2_) analyzed for gene-of-interest expression.

### Genome walking

Physical mapping of the transgene insertion site in line P4 was performed by genome walking using the Clontech Universal Genome Walker kit (Takara Bio, Mountain View, CA) [40]. Total genomic DNA was extracted from eight transgenic larvae of line P4 using the Pure Gene DNA extraction kit (Qiagen, Valencia, CA). Extracted DNA was digested with *Eco*RV and *Stu*I, followed by phenol-chloroform extraction and ligation to the universal genome walker adapter provided with the kit. The ligated products were subjected to a first round of PCR amplification using the following sets of primers: left integration site gene-specific primer forward (left GSP1 F; S1 Table) and adaptor primer 1 (AP1); right GSP1 R (S1 Table) and AP1. The first-round PCR amplification products became the templates for a second round of PCR amplification using nested gene specific primers: left GSP2 F (S1 Table) and AP2; right GSP2 R (S1 Table) and AP2. The PCR amplified products were column purified (Promega, Madison, WI) and cloned into TOPO 4.1 vector (Invitrogen, Carlsbad, CA) for Sanger sequencing. The gene insertion locus was mapped to the *Ae*. *aegypti* genome (VectorBase AaegL3 assembly) and confirmed by PCR amplification using genome locus specific primers (S1 Table).

### Generation of a recombinant CHIKV expressing AeTIMP_V5_

The *Aetimp*_V5_ coding sequence was PCR amplified from midgut cDNA libraries as described above, using forward and reverse primers containing *Asc*I and *Pme*I restriction sites. The PCR product was gel purified and inserted into the *Asc*I/*Pme*I digested full-length infectious cDNA clone of CHIKV (strain: LR2006-OPY1; [40]), which harbored the A226V amino acid change in E1 [56,57]. The viral cDNA clone was engineered to have its duplicated subgenomic promoter for gene-of-interest expression positioned upstream of the structural protein encoding genes [38]. The resulting pCHIKV-AeTIMP_V5_ construct was linearized overnight with *Not*I. Linearized plasmid (2 μg) was *in vitro* transcribed for 4 hours using the MEGAscript SP6 transcription kit (ThermoFisher Waltham, MA). BHK21 cells were seeded into a six-well plate at a density of 2 x 10^5^ cells/well and incubated for 2 days to obtain 80% confluency. Lipofectamine LTX reagent (3%; Invitrogen) was added to 100 μl of serum-free DMEM and mixed with 15 μl PLUS transfection reagent (prep. 1). Simultaneously, 6 μl of *in vitro* transcribed RNA was diluted in 100 μl of serum-free DMEM (prep. 2). After 10 minutes of separate incubations, both preparations were mixed together and allowed to incubate for 20 minutes before transfection. After transfection with CHIKV-AeTIMP_V5_ RNA, cells were observed daily for CPE development, indicating viral replication. Cell supernatant was collected on day 3 post-transfection and virus titer determined by plaque assay in Vero cells (#CCL-81, American Type Culture Collection, Manassas, VA). The same procedure was followed to generate CHIKV-EGFP.

### Recombinant TIMP protein expression and purification

The *Aetimp* coding sequence was PCR amplified using forward and reverse primers containing *Hind*III and *Xho*I restriction sites, respectively (S1 Table). The resulting amplicon was cloned into the pSecTag2 expression plasmid (Invitrogen). The resulting recombinant pSec/Aetimp plasmid was transfected into HEK293T cells using LipoD293 DNA *In Vitro* Transfection Reagent (Signagen, Rockville MD). Control cells were transfected with the pSecTag2 (blank) vector. Twenty-four hours post-transfection, the FBS containing DMEM cell culture medium was replaced with fresh serum-free medium. Two days later, the protein containing medium was collected and centrifuged at 3,000 *g* to remove cell debris, then stored at -80°C. Proteins were separated under reducing conditions in a 12% SDS-PAGE and recombinant proteins were detected by Western blot using a monoclonal anti-6×His-tag specific antibody (ThermoFisher Scientific). To purify recombinant proteins, cell-free medium (30 ml) was incubated under continuous rotation for 1h at 4°C with 2 ml of Ni-NTA His-bind resin (Thermo Scientific), then equilibrated with binding buffer (20 mM sodium phosphate, pH 7.4, 0.3 M sodium chloride, 10 mM imidazole). Ni-NTA His-bind resin and medium were packed into an empty spin column (9 cm high and 2 ml bed volume; Bio-Rad, Hercules, CA) placed on top of a 15 ml tube that was sitting on ice. The column was washed with 60 ml of binding buffer. Bound proteins were eluted in four subsequent, separately collected eluates, each one containing 0.5 ml of elution buffer (20 mM sodium phosphate, pH 7.4, 0.3 M sodium chloride, 250 mM imidazole). The eluates were analyzed by SDS-PAGE and Western blot using a monoclonal anti-6×His-tag antibody. Fractions containing recombinant protein were then equilibrated and concentrated using Centrifugal Filter Units (10 kDa cut off; Millipore, Billerica, MA).

### Recombinant AeMMP expression

Recombinant AeMMP1 and AeMMP2 proteins were expressed in *Drosophila* S2 cells. *Aemmp1* and *Aemmp2* coding sequences (lacking the signal peptide sequences) were PCR amplified with forward and reverse primers containing *Spe*I and *Xho*I restriction sites, respectively (S1 Table). Amplicons were inserted into expression plasmid vector pMT/Bip/V5-His B (Invitrogen) that was modified by having the encoded His tag removed. The resulting recombinant pMT/AeMMP1/V5 and pMT/AeMMP2/V5 plasmids were transiently transfected into S2 cells using TransFectin Lipid Reagent (Bio-Rad) following the standard protocol. Control cells were transfected with the (modified) pMT/Bip/V5 (blank) vector. Recombinant protein production was induced with 500 μM CuSO_4_ in serum-free Schneider's Insect Medium (Lonza, Basel, Switzerland). Protein containing medium was collected at 3 days after induction. Recombinant AeMMPs were detected by Western blot using AeMMP specific antibodies [27]. S2 cell generated AeMMP proteins were also used for *in vitro* activity assays.

### Arbovirus infections of mosquitoes and virus detection in mosquito tissues

The propagation of CHIKV (strain: 37997), infection of mosquitoes via CHIKV containing bloodmeals, and CHIKV detection by plaque assays have been previously described [7,27]. Intrathoracic injection of CHIKV into HWE females was performed using the Nanoject II injection system (Drummond Scientific, Broomall, PA). Each mosquito received ~200 nl of 10^5^ pfu/ml virons.

Dengue virus type 4 (DENV4, strain: H-241, Philippines) was propagated in *Ae*. *albopictus* C6/36 cells in T25 flasks at a multiplicity of infection (m.o.i.) of 0.01 using Leibovitz's L-15 modified media (Corning Mediatech Inc., Manassas, VA) complemented with 2% FBS (Sigma) and 1% non-essential amino acids (Corning Mediatech Inc.). After 4–5 days incubation at 28°C, infected cell culture medium was mixed with an equal volume of defibrinated sheep blood containing 10mM ATP. One week-old females were fed for 1h with virus-infected cell culture-blood mixture at 37°C using a single glass feeder per carton. Fully engorged females were selected and maintained on raisins and water until further analysis. At 7 and 14 dpi, mosquitoes were collected and processed for plaque assays as described earlier using BHK21 cells [40]. All virus infections of mosquitoes and virus detection assays were carried out in a Biosafety Level 3 laboratory within the Laboratory for Infectious Disease Research (LIDR) at the University of Missouri.

### Immunofluorescence assays and Western blot analyses

A group of five midguts was fixed in 4% p-formaldehyde (Sigma) for 1 day up to one week at 4°C. After three washes with PBS, samples were permeabilized by incubation with PBT (PBS containing 1% BSA and 0.2% Triton X-100) for 1h on a rocker at room temperature (RT). Samples were then incubated overnight at 4°C with anti-V5 tag mouse monoclonal antibody (Invitrogen) at a dilution of 1:2000 in PBT or anti-CHIKV mouse monoclonal antibody (Abcam #B1414; Cambridge, MA, USA) at a dilution of 1:100. After washing four times with 200μl PBST (0.1% Triton X-100), samples were incubated for 1.5h with 100μl 1:200 anti-mouse IgG Alexa Fluor 488 (Cell Signaling Technology, Danvers, MA) in PBT at 37°C in the dark. DAPI (Invitrogen) was added to each sample to stain nuclei. Individual midgut samples were placed on glass slides, mounted with Fluoromount-G (Electron Microscopy Sciences Hatfield, PA), and viewed under an inverted spectral confocal microscope (TCP SP8 MP, Leica Microsystems) located at the Molecular Cytology Core of the University of Missouri.

For Western blots, midguts were homogenized in 2x Laemmli sample buffer (Bio-Rad), boiled for 5 min, and centrifuged at 10,000 *g* for 10 min. The supernatants were separated by SDS-PAGE and transferred to a nitrocellulose membrane. After blocking with 5% non-fat dried milk in Tris-buffered saline (20 mM Tris-HCl, 150 mM NaCl, 1 mM EDTA, 0.1% Tween 20, pH 7.5) (TBST) for 1 h, the membrane was incubated in the blocking solution overnight at 4°C with anti-V5 tag monoclonal antibody, monoclonal anti-His-tag antibody (Thermo Scientific), human anti-collagen IV polyclonal antibodies (Abcam #ab6586), or polyclonal antibodies pAb-mmp1-1 and pAb-mmp1-2 [27]. Following overnight incubation with the primary antibody, membranes were washed three times (10 min/wash) in TBST. The membranes were incubated with anti-rabbit IgG-HRP or anti-mouse IgG-HRP (Cell Signaling Technology) at RT for 2 h and then treated for 1 min with SuperSignal West Pico chemiluminescent substrate (Pierce, Waltham, MA). The immuno-reactive proteins were visualized by exposing the membrane to an x-ray film. Anti-β-actin-peroxidase antibody (Sigma) was used to detect the loading control in each lane.

### dsRNA synthesis and mosquito injections

For each target gene, DNA templates of around 500 bp were amplified from midgut cDNA using forward and reverse primers encoding T7 promoter sequences at their 5' ends. Using the MEGAscript T7 kit (Ambion, Austin, TX), 1 μg of template cDNA was *in vitro* transcribed at 37°C for 4 h to generate dsRNA. The generated dsRNA was treated with turbo DNAse I at 37°C for 15 min, purified using the MEGAclear kit (Ambion), and adjusted with nuclease-free water to a final concentration of 2 μg/μl. Around 140 ng dsRNA was injected intrathoracically into one-week old females using the Nanoject II injection system (Drummond Scientific). Two days after dsRNA injection, mosquitoes received a CHIKV-containing or a non-infectious bloodmeal. Midguts and carcasses were dissected and processed for qRT-PCR and/or plaque assays as described before [7,27] using specific primers listed in S1 Table.

### Administering MMP and collagenase inhibitors to mosquitoes

Female mosquitoes received bloodmeals consisting of the supernatant of CHIKV infected (~10^7^ pfu/ml) cell culture, 1:1 mixed with defibrinated sheep blood and supplemented with either a) MMP inhibitor GM6001 at 0.1 mM or 1 mM final concentrations; b) MMP inhibitor Batimastat BB-94 (Sigma) at 0.1 mM or 1 mM final concentrations, or c) collagenase inhibitor I (Millipore) at 0.5 mM final concentration. In the control, the MMP inhibitor was substituted for DMSO. GM6001 at concentrations of 0.1 mM or 0.5 mM was also intrathoracically injected into females using the Nanoject II injection system (Drummond Scientific). One and two days following feeding of inhibitor containing bloodmeals (or GM6001 injection), midguts were dissected to monitor collagen IV degradation by Western blot analysis. Carcasses were collected at 2 and 4 dpi and processed for plaque assays in Vero cells.

### Enzymatic assays

#### Collagen digestion *in vitro* assay

Five μg of collagen I from human placenta (Sigma) were incubated with 1 μl of midgut lysate (equivalent to 1/5 midgut) at 28°C for 18 h. Midgut lysates were prepared by homogenizing 10 midguts in 50 μl assay buffer (50 mM Tris-HCl, 150 mM NaCl, 5 mM CaCl_2_, pH 7.6), and collecting supernatants after centrifugation at 10,000 *g* for 10 min at 4°C. Following incubation, the collagen I/midgut lysate mixture was analyzed on a 10% SDS-PAGE gel under reducing condition. The protein bands were visualized by staining with Coomassie blue G-250 (Bio-Rad).

#### Zymography

Protein containing, conditioned medium was mixed with 4x SDS sample buffer (Bio-Rad) and separated under non-reducing conditions in a 10% SDS-PAGE containing 1 mg/ml gelatin (Sigma). The gel was then washed twice in 100 ml of washing buffer (2.5% (v/v) Triton X-100 in water) and incubated in 100 ml of assay buffer (20 mM Tris/HCl, pH 7.5, 1.25% (v/v) Triton X-100, 5 mM CaCl_2_) for ~20 h at 37°C. Gels were stained with 0.2% Coomassie brilliant blue R-250 (in 30% methanol and 10% acetic acid) and destained in a solution containing 30% methanol and 10% acetic acid. Gelatinase activity became evident by the appearance of cleared regions on the gel.

#### rMMP activity assays and rTIMP inhibition

HuMMP1-3 were purchased from Anaspec (Fremont, CA). HuMMP1 and HuMMP2 were activated via incubation with 1 mM APMA at 37°C for 3 h and 1 h, respectively. Twenty ng of activated HuMMPs were then incubated with 4 ng or 20 ng of AeTIMP for 2 h at room temperature. In the AeTIMP inhibition assay for HuMMP2, DQ gelatin (Molecular Probes, Eugene, OR) was added to a final concentration of 50 μg/mL to the reaction buffer (50 mM Tris-HCl, 150 mM NaCl, 5 mM CaCl_2_, pH 7.6) and fluorescence intensities were measured at Ex = 495 nm and Em = 515 nm every 20 min (for 2 h) with a Perkin-Elmer LS50B spectrometer. In the AeTIMP inhibition assays for HuMMP1 and HuMMP3, FS-6 (Millipore) was added to a final concentration of 10 μM to the reaction buffer. Fluorescence intensities were measured at Ex = 328 nm and Em = 393 nm. AeMMP activities in midgut preparations were measured using the Generic MMP Assay Kit (Anaspec) and/or the DQ collagen IV substrate (Molecular Probes) according to the protocols provided. Each sample consisted of three independent biological replicates.

### Statistical analyses

Statistical analysis was performed using the GraphPad Prism software package (version 6.01). CHIKV titers were compared using the non-parametric Mann-Whitney U-test. Prevalence of CHIKV infection in mosquito tissues was analyzed using Fisher's exact test. Data from the qRT-PCR experiments and *in vitro* activity assays were analyzed using one-way analysis of variance (ANOVA) followed by Tukey's multiple comparisons test or Student’s *t*-test. All tests were considered significant at *p* ≤ 0.05.

### Accession numbers

CHIKV LR2006-OPY1: GenBank KT449801.1; CHIKV 37997: GenBank AY726732.1; *Ae*. *aegypti* TIMP (AeTIMP): VectorBase AAEL013525; *Ae*. *albopictus* TIMP (AaTIMP): VectorBase AALF008324/AALF025384; human TIMP3 (HuTIMP3): GenBank NM000362.

## Supporting information

S1 Table Primers used for experiments. (DOCX)

S1 Fig Supplementation with proteinase inhibitors did not affect midgut collagen IV abundance and CHIKV dissemination from the midgut. **(A)** Detection of collagen IV by Western blot in midguts of mosquitoes that had received bloodmeals containing different proteinase inhibitors at 1 days pbm: GM6001 (MMP inhibitor), BB-94 (batimastat), CI (collagenase inhibitor). **(B)** Detection of collagen IV by Western blot in midguts of mosquitoes that were bloodfed following intrathoracic injection with different concentrations of MMP inhibitor GM6001 or DMSO (control). Samples were collected at 1, 2, and 4 days pbm. **(C)** Detection of collagen IV by Western blot in midguts of mosquitoes that had received bloodmeals containing GM6001 or DMSO (control). Collagen IV was detected using polyclonal antibodies generated against human collagen IV. **(D)** CHIKV titers in individual carcasses of mosquitoes that had received bloodmeals containing 10^7^ pfu/ml CHIKV supplemented with either GM6001 or DMSO (control) at 2 and 4 dpi as detected by plaque assays in Vero cells. Images shown in panels A, B, C, and graph of panel D are representative examples of repeated experiments.
(TIF)

S2 Fig Mapping of binding sites of polyclonal antibodies pAb-mmp1-1 and pAb-mmp1-2 to functional domains of AeMMP1. The image was adapted from NCBI Protein Blast: conserved domains graphical summary for AAEL005666-PA.
(TIF)

S3 Fig Transient silencing of *Aemmp1* did not affect AeMMP1 protein abundance, midgut collagen IV abundance, and CHIKV dissemination from the midgut. **(A)** qRT-PCR detection of *Aemmp1* expression in whole-body mosquitoes at 2 days following dsRNA injection. Statistical analysis was performed using Student’s *t*-test (*p* ≤ 0.05). Detection of **(B)** AeMMP1 and **(C)** collagen IV by Western blot in midguts of *luciferase* dsRNA (negative control) and *Aemmp1* dsRNA injected mosquitoes at 24 h pbm (= 3 days post-dsRNA injection). aMMP1 = catalytically active form of AeMMP1. Control: midguts of non-injected mosquitoes, which had received a bloodmeal; sugar: midguts of non-injected mosquitoes fed on sugar. **(D)** CHIKV titers in individual carcasses of dsRNA injected mosquitoes at 2 dpi (dsRNA injections were performed 2 days before oral virus challenge). Statistical analysis was performed using the Mann-Whitney U-test (* at *p* ≤ 0.05).
(TIF)

S4 Fig Mosquito TIMPs and HuTIMP3 share conserved amino acid motifs and inhibit/reduce MMP activities *in vitro*. **(A)** Amino acid sequence alignment of TIMP proteins from *Ae*. *aegypti* (AeTIMP), *Ae*. *albopictus* (AaTIMP), and human TIMP3 (HuTIMP3). In red: conserved cysteine residues potentially involved in disulfide bonding; in bold and black: amino acid residues that differ between AeTIMP and AaTIMP. The dark blue line shows the demarcation of the N-terminal and C-terminal subdomains. **(B)** Kinetics of HuMMP2 and **(C)** HuMMP3 activities and their inhibition by HuTIMP3 *in vitro* using FS-6 as substrate. Twenty ng of HuMMP3 were preincubated with 20 ng of HuTIMP3 or buffer at RT for 2 h, followed by addition of FS-6. Fluorescence intensity was measured every 20 min. **(D)** Kinetics of rAeTIMP-mediated inhibition of rAeMMP1. Four ng or 20 ng of rAeTIMP, were incubated with 20 ng of rAeMMP at RT for 2 h, followed by addition of FS-6 substrate and incubation for an additional 2–4 h. rAeTIMP was also incubated in absence of rAeMMP1 to demonstrate that rAeTIMP alone was unable to cleave the substrate. Fluorescence intensity was measured every 20 min.
(TIF)

S5 Fig Transient silencing of AeTIMP did not affect CHIKV dissemination efficiency. **(A)** qRT-PCR detection of *Aetimp* expression in whole-body mosquitoes, which had been injected with *Aetimp* dsRNA, *egfp* dsRNA, or PBS. At 2 days post-dsRNA injection, total RNA was extracted from sugarfed mosquitoes and used for qRT-PCR assays. Another group of mosquitoes received a bloodmeal at 2 days post-dsRNA injection and total RNA was extracted at 2 days pbm. Statistical analysis was performed using one-way analysis of variance (ANOVA) followed by Tukey's multiple comparisons test (*p* ≤ 0.05). **(B)** CHIKV titers in carcasses of mosquitoes at 2 dpi, which had been injected with *Aetimp* dsRNA, *egfp* dsRNA, or PBS 2 days before virus infection. Each data point represents the CHIKV titer of an individual carcass. *P*-values were determined by the Mann-Whitney U-test. Black bars indicate medians.
(TIF)

S6 Fig Genotypic and phenotypic analyses of transgenic P4 mosquitoes. **(A)** Integration of the *mariner Mos1* TE into the genome of P4 mosquitoes. A single integration event in supercontig 1.342 at nt position 1,211,152 (chromosome 2q) was revealed. Bold and highlighted in red: TA recognition motif for *mariner Mos1* in the genome of HWE where TE integration took place. Highlighted in green: right arm of the TE; highlighted in blue: left arm of the TE. Bold and black: TA target site duplication. **(B)** Immunofluorescence assay showing presence of CHIKV antigen (green) in midguts of HWE and **(C)** P4 mosquitoes. Viral antigen was detected using a CHIKV-specific monoclonal antibody. Nuclei were stained with DAPI (blue).
(TIF)

S7 Fig Ultrastructural imaging of the midgut surfaces of bloodfed HWE and transgenic P4 mosquitoes. Left image: HWE, 24 h post-bloodmeal; right image: P4, 24 h post-bloodmeal. Images were captured with a FEI Quanta 600F scanning electron microscope.
(TIF)
