# Supplementary material for: Chikungunya virus dissemination from the midgut of Aedes aegypti is associated with temporal basal lamina degradation during bloodmeal digestion
Source: PLoS Negl Trop Dis. 2017 Sep 29;11(9):e0005976. doi: 10.1371/journal.pntd.0005976 (PMC5636170; doi:10.1371/journal.pntd.0005976)
Supplement: S1 Table — (DOCX) [file pntd.0005976.s001.docx]

| **Purpose** | **Name** | **Sequence (5’ to 3’)** |
| --- | --- | --- |
| qPCR | qTIMP F | GCGCAAGTACAGCGATCTTA |
|  | qTIMP R | GCACGATCCGTAATCTGTCTC |
|  | qMMP1 F | CAAACAACGTCACAAGCAGAGC |
|  | qMMP1 R | AGCTCTGGAACTCCATGATTGC |
|  | qMMP2 F | CAACTCGCAACATTTGGAACC |
|  | qMMP2 R | CGTCTTCGTCGAATCTCCAATAA |
|  | rpS7 F | GGAGAAGAAGTTCTCCGGCAAG |
|  | rpS7 R | TGAAGGTGTCGACCTTGTGTTC |
| (Semi-) RT-PCR | TIMP check F | GAACCGAAAGCATAACGACAATAAC |
|  | TIMP check R | CACGATCCGTAATCTGTCTCAC |
|  | V5 check R | GGAGAGGGTTAGGGATAGGCTT |
|  | Actin F | AAGGCCAACCGTGAGAAGATGACT |
|  | Actin R | GCTCGTTGCCAATGGTGATGAC |
| dsRNA | MMP1 T7 F | GTAATACGACTCACTATAGGGCGGAGAATGCCGTGGCCG |
|  | MMP1 T7 R | GTAATACGACTCACTATAGGGCACTGGTGGTACGGCTGCAAG |
|  | TIMP T7 F | GTAATACGACTCACTATAGGGATGTTACAAACGAGAATGAATC |
|  | TIMP T7 R | GTAATACGACTCACTATAGGGGTCCACTTTACACTTTTGATAG |
|  | egfp T7 F | GTAATACGACTCACTATAGGGTGACCCTGAAGTTCATCTGCACCAC |
|  | egfp T7 R | GTAATACGACTCACTATAGGGCTCCAGCAGGACCATGTGATCGCG |
| Transgenic lines | TIMP NotI f | TAGCACGTAGCGGCCGCATGTTACAAACGAGAATGAATC |
|  | TIMP V5 SacII R | TAACCGCGGTCACGTAGAATCGAGACCGAGGAGAGGGTTAGGGATAGGCTTACCTTCTCTTGTGTCCACTTTAC |
| Genome walking | GSP1 F | CAATTATGACGCTCAATTCGCGCCAAAC |
|  | GSP1 R | GAGCAGCGCTTCGATTCTTACGAAAGTGTG |
|  | GSP2 F | GTGGTTCGACAGTCAAGGTTGACACTTC |
|  | GSP2 R | GACGATGAGTTCTACTGGCGTGGAATCC |
| Recombinant CHIKV | TIMP F Asc I | ATCGTAGCAGGCGCGCCATGTTACAAACGAGAATG |
|  | TIMP V5 Pme I R | ACTGGCACGGTTTAAACTCACGTAGAATCGAGACCGAGGAG |
| Recombinant protein | TIMP HindIII F | ATCAAGCTTGATGTTACAAACGAGAATGAATCC |
|  | TIMP XhoI R | TAACTCGAGATTCTCTTGTGTCCACTTTACAC |
|  | MMP1 SpeI F | ATCACTAGTCCTGTCCAAACAACGTCACA |
|  | MMP1 Xho R | TAACTCGAGGGCTGATTGGAATTTTAGTAC |
|  | MMP2 SpeI F | ATCACTAGTATGACCGATCTAGATGCGG |
|  | MMP2 Xho R | TAACCTCGAGACCACCTATTTCGAAGGTTC |

**S1 Table: Primers used for experiments**
