# Supplementary figures and images for: Chikungunya virus dissemination from the midgut of Aedes aegypti is associated with temporal basal lamina degradation during bloodmeal digestion
Source: PLoS Negl Trop Dis. 2017 Sep 29;11(9):e0005976. doi: 10.1371/journal.pntd.0005976 (PMC5636170; doi:10.1371/journal.pntd.0005976)

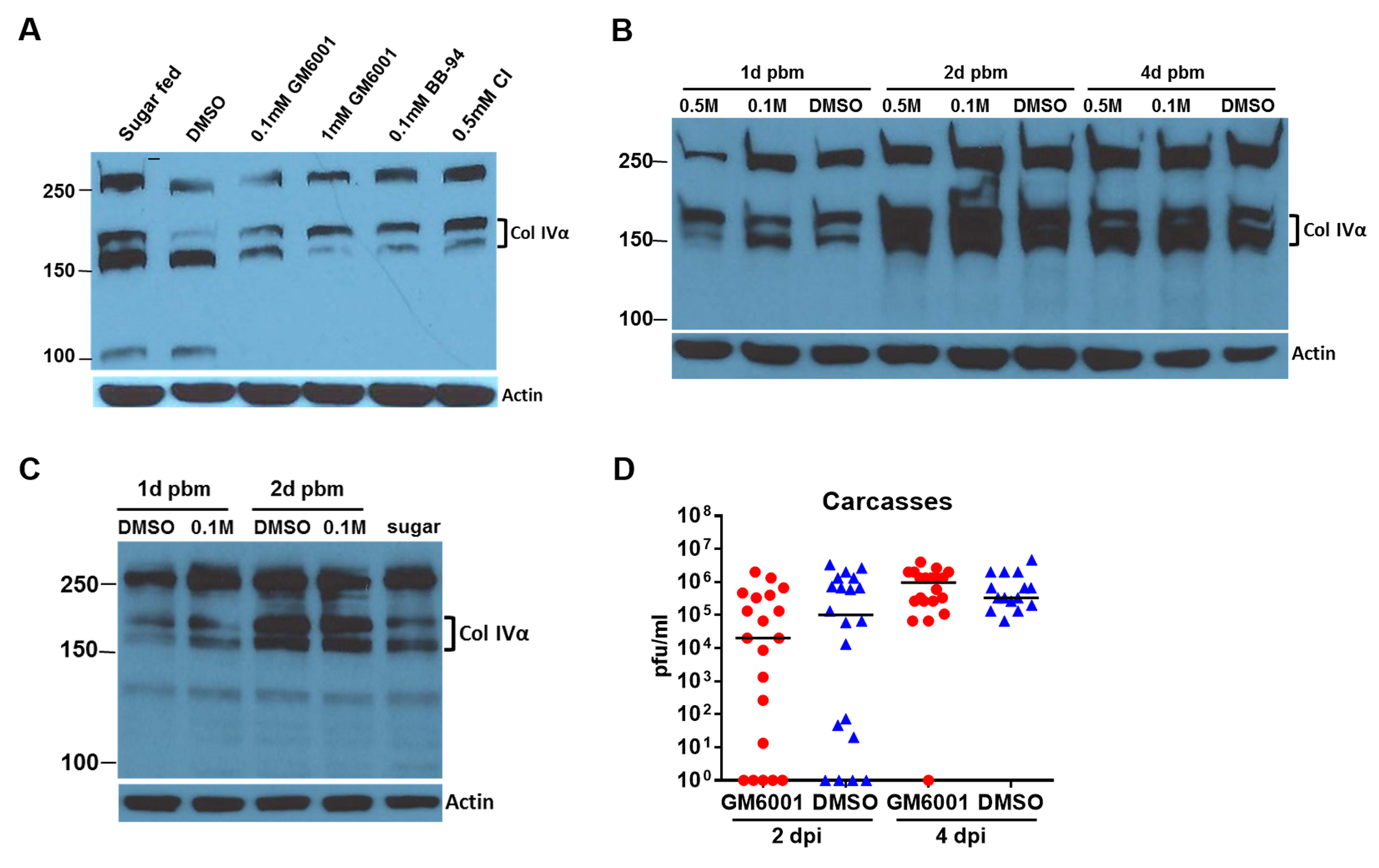

Supplement: S1 Fig — (A) Detection of collagen IV by Western blot in midguts of mosquitoes that had received bloodmeals containing different proteinase inhibitors at 1 days pbm: GM6001 (MMP inhibitor), BB-94 (batimastat), CI (collagenase inhibitor). (B) Detection of collagen IV by Western blot in midguts of mosquitoes that were bloodfed following intrathoracic injection with different concentrations of MMP inhibitor GM6001 or DMSO (control). Samples were collected at 1, 2, and 4 days pbm. (C) Detection of collagen IV by Western blot in midguts of mosquitoes that had received bloodmeals containing GM6001 or DMSO (control). Collagen IV was detected using polyclonal antibodies generated against human collagen IV. (D) CHIKV titers in individual carcasses of mosquitoes that had received bloodmeals containing 107 pfu/ml CHIKV supplemented with either GM6001 or DMSO (control) at 2 and 4 dpi as detected by plaque assays in Vero cells. Images shown in panels A, B, C, and graph of panel D are representative examples of repeated experiments. (TIF) [file pntd.0005976.s002.tif]

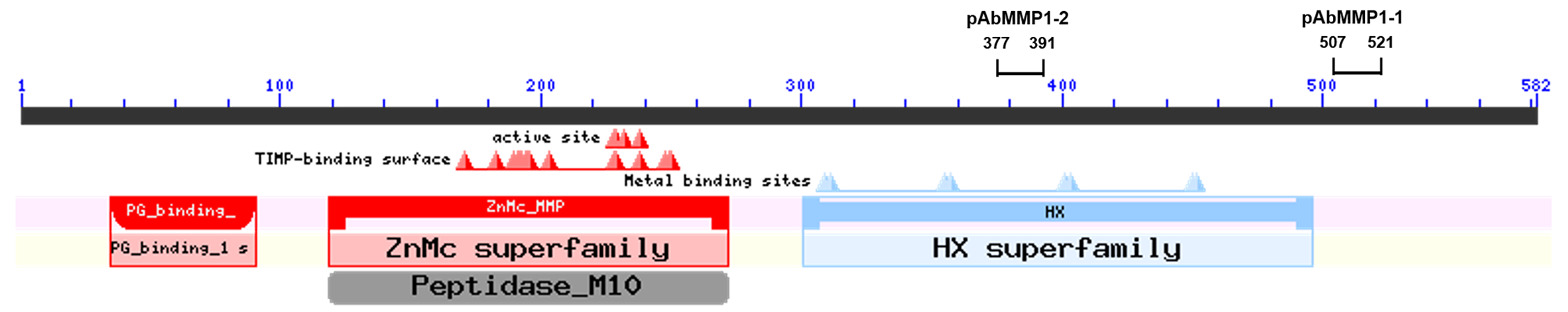

Supplement: S2 Fig — The image was adapted from NCBI Protein Blast: conserved domains graphical summary for AAEL005666-PA. (TIF) [file pntd.0005976.s003.tif]

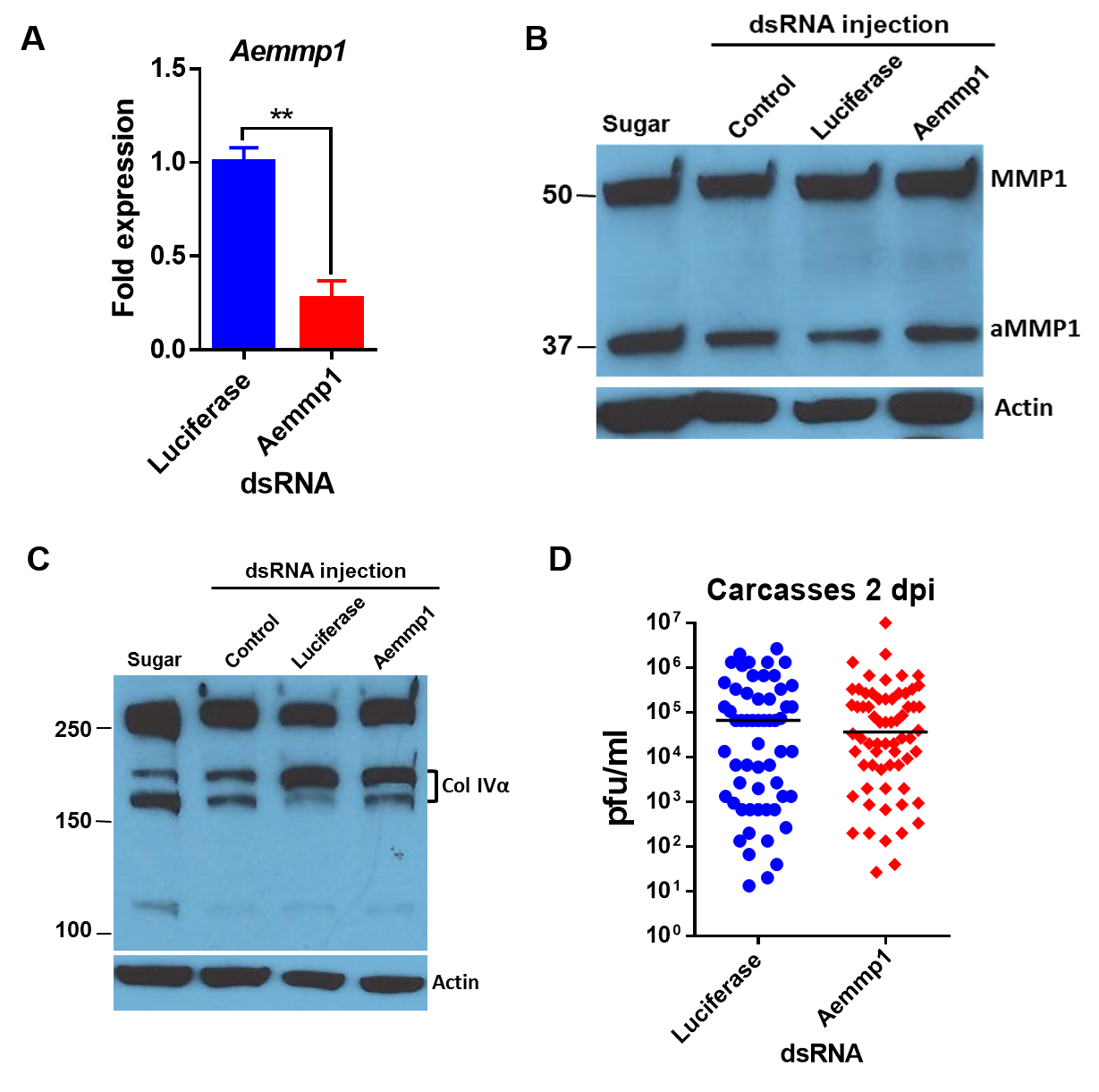

Supplement: S3 Fig — (A) qRT-PCR detection of Aemmp1 expression in whole-body mosquitoes at 2 days following dsRNA injection. Statistical analysis was performed using Student’s t-test (p ≤ 0.05). Detection of (B) AeMMP1 and (C) collagen IV by Western blot in midguts of luciferase dsRNA (negative control) and Aemmp1 dsRNA injected mosquitoes at 24 h pbm (= 3 days post-dsRNA injection). aMMP1 = catalytically active form of AeMMP1. Control: midguts of non-injected mosquitoes, which had received a bloodmeal; sugar: midguts of non-injected mosquitoes fed on sugar. (D) CHIKV titers in individual carcasses of dsRNA injected mosquitoes at 2 dpi (dsRNA injections were performed 2 days before oral virus challenge). Statistical analysis was performed using the Mann-Whitney U-test (* at p ≤ 0.05). (TIF) [file pntd.0005976.s004.tif]

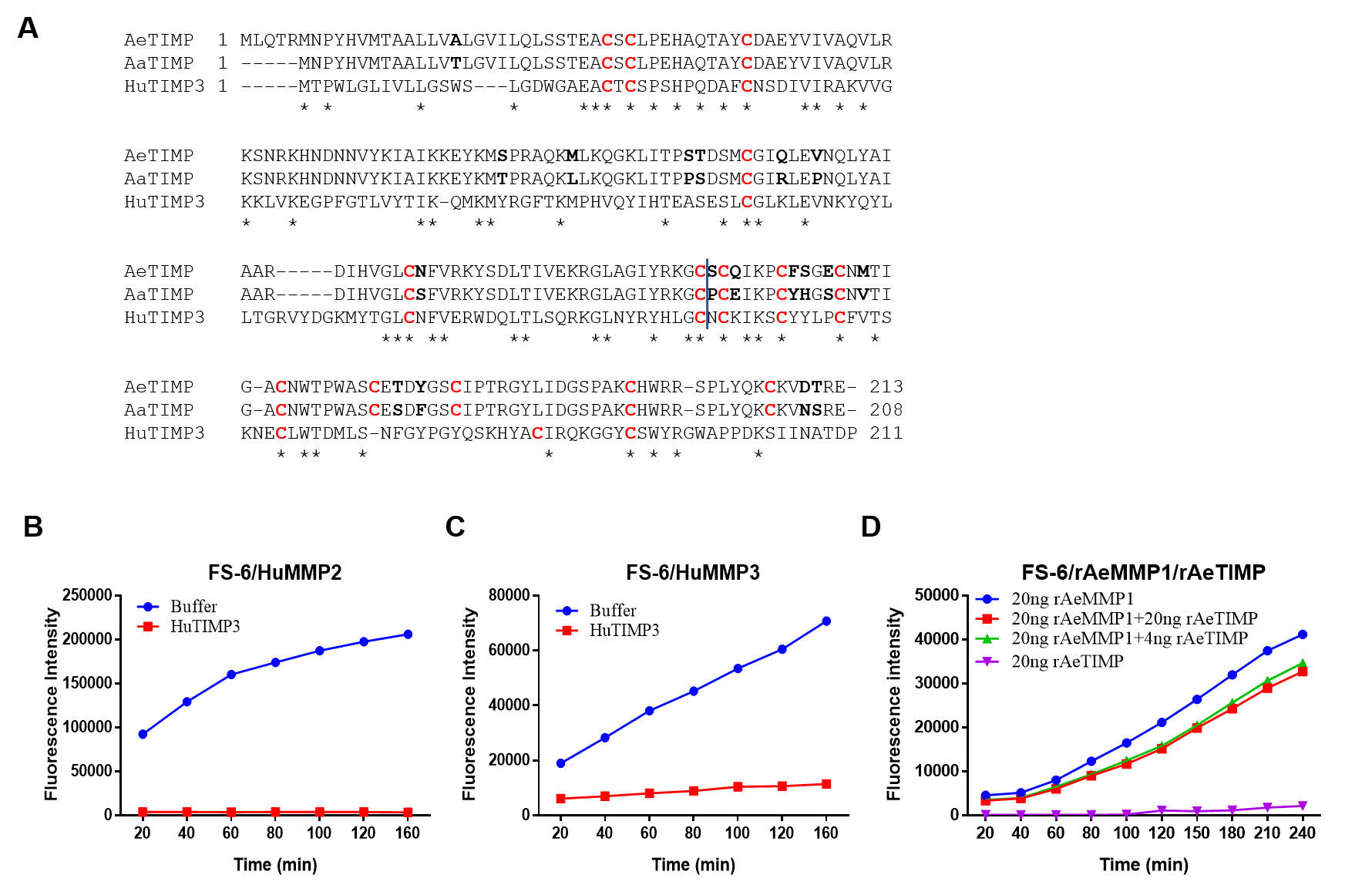

Supplement: S4 Fig — (A) Amino acid sequence alignment of TIMP proteins from Ae. aegypti (AeTIMP), Ae. albopictus (AaTIMP), and human TIMP3 (HuTIMP3). In red: conserved cysteine residues potentially involved in disulfide bonding; in bold and black: amino acid residues that differ between AeTIMP and AaTIMP. The dark blue line shows the demarcation of the N-terminal and C-terminal subdomains. (B) Kinetics of HuMMP2 and (C) HuMMP3 activities and their inhibition by HuTIMP3 in vitro using FS-6 as substrate. Twenty ng of HuMMP3 were preincubated with 20 ng of HuTIMP3 or buffer at RT for 2 h, followed by addition of FS-6. Fluorescence intensity was measured every 20 min. (D) Kinetics of rAeTIMP-mediated inhibition of rAeMMP1. Four ng or 20 ng of rAeTIMP, were incubated with 20 ng of rAeMMP at RT for 2 h, followed by addition of FS-6 substrate and incubation for an additional 2–4 h. rAeTIMP was also incubated in absence of rAeMMP1 to demonstrate that rAeTIMP alone was unable to cleave the substrate. Fluorescence intensity was measured every 20 min. (TIF) [file pntd.0005976.s005.tif]

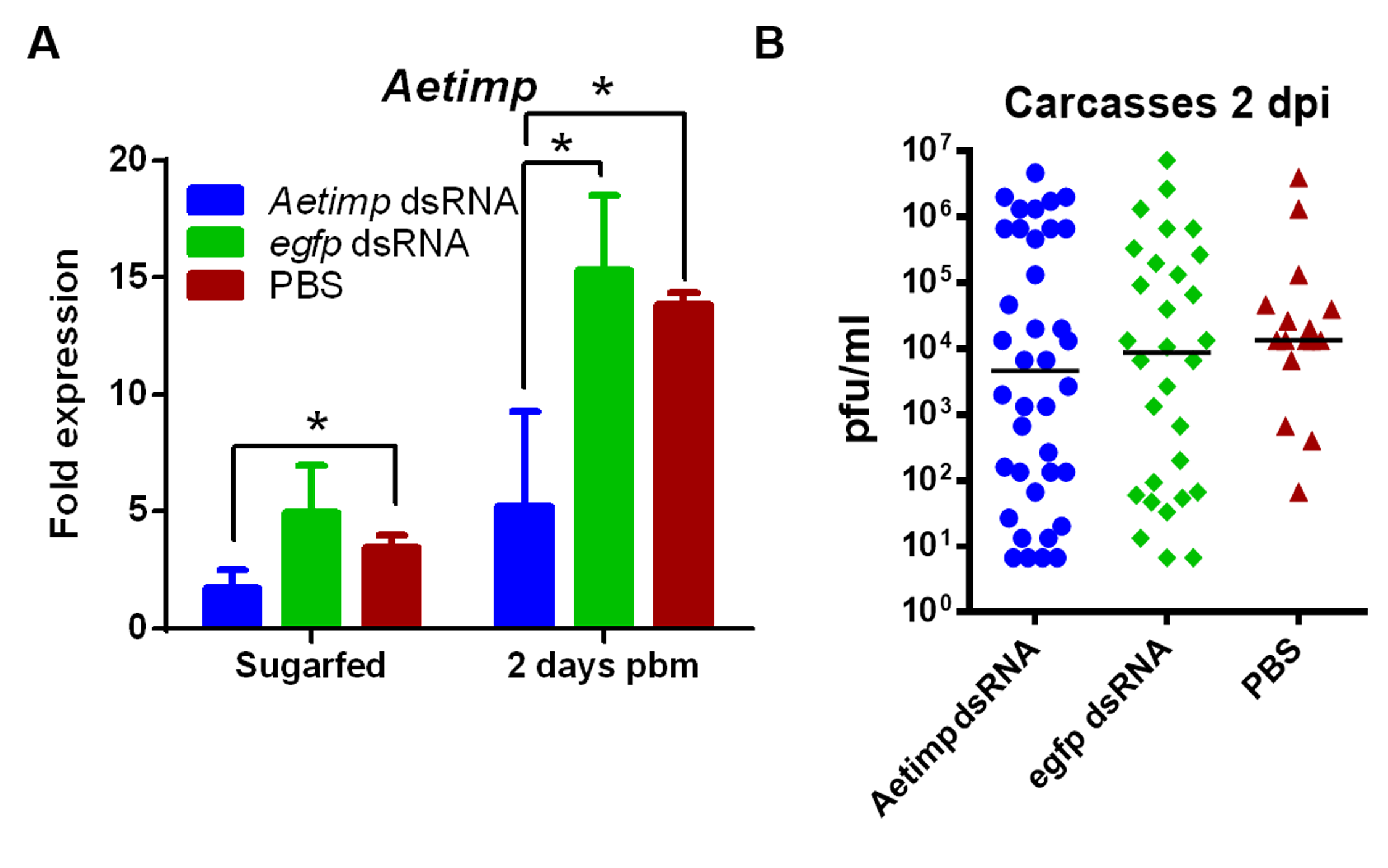

Supplement: S5 Fig — (A) qRT-PCR detection of Aetimp expression in whole-body mosquitoes, which had been injected with Aetimp dsRNA, egfp dsRNA, or PBS. At 2 days post-dsRNA injection, total RNA was extracted from sugarfed mosquitoes and used for qRT-PCR assays. Another group of mosquitoes received a bloodmeal at 2 days post-dsRNA injection and total RNA was extracted at 2 days pbm. Statistical analysis was performed using one-way analysis of variance (ANOVA) followed by Tukey's multiple comparisons test (p ≤ 0.05). (B) CHIKV titers in carcasses of mosquitoes at 2 dpi, which had been injected with Aetimp dsRNA, egfp dsRNA, or PBS 2 days before virus infection. Each data point represents the CHIKV titer of an individual carcass. P-values were determined by the Mann-Whitney U-test. Black bars indicate medians. (TIF) [file pntd.0005976.s006.tif]

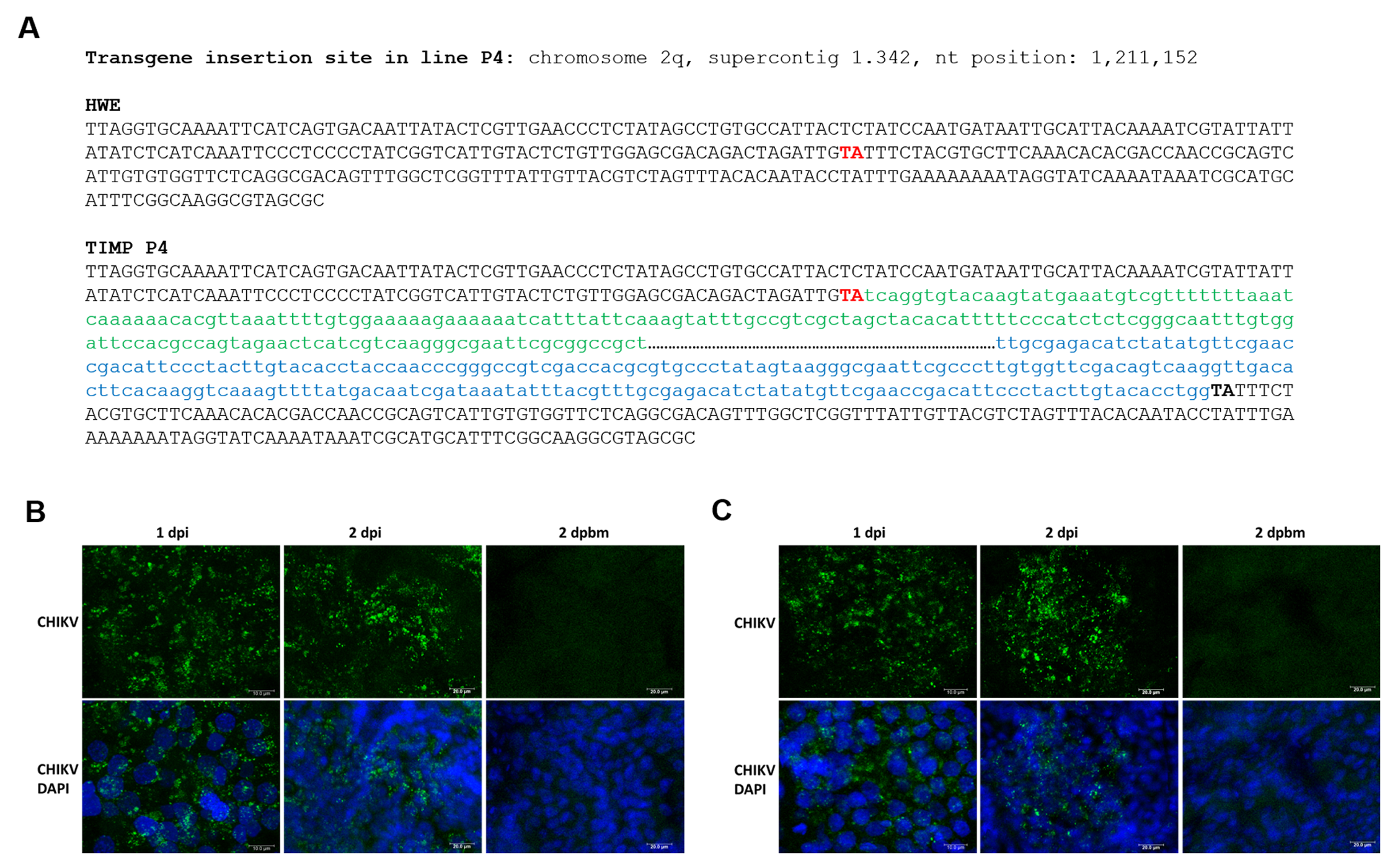

Supplement: S6 Fig — (A) Integration of the mariner Mos1 TE into the genome of P4 mosquitoes. A single integration event in supercontig 1.342 at nt position 1,211,152 (chromosome 2q) was revealed. Bold and highlighted in red: TA recognition motif for mariner Mos1 in the genome of HWE where TE integration took place. Highlighted in green: right arm of the TE; highlighted in blue: left arm of the TE. Bold and black: TA target site duplication. (B) Immunofluorescence assay showing presence of CHIKV antigen (green) in midguts of HWE and (C) P4 mosquitoes. Viral antigen was detected using a CHIKV-specific monoclonal antibody. Nuclei were stained with DAPI (blue). (TIF) [file pntd.0005976.s007.tif]

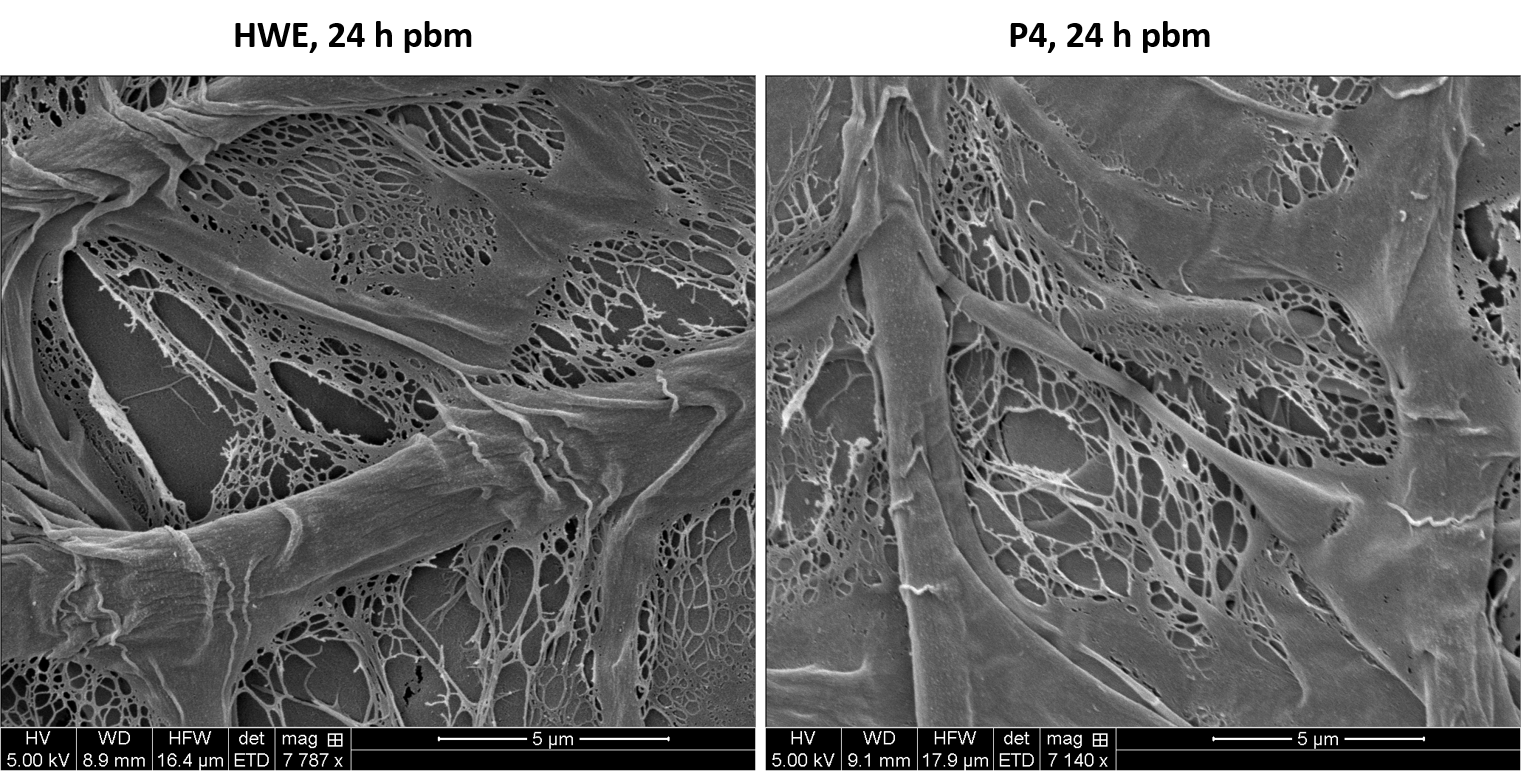

Supplement: S7 Fig — Left image: HWE, 24 h post-bloodmeal; right image: P4, 24 h post-bloodmeal. Images were captured with a FEI Quanta 600F scanning electron microscope. (TIF) [file pntd.0005976.s008.tif]
